# Chiropractic spinal manipulative therapy versus physical therapist‐led exercise and the risk of cauda equina syndrome in adults with lumbar disc herniation, stenosis, or radiculopathy

**DOI:** 10.1002/pmrj.70071

**Published:** 2026-01-03

**Authors:** Robert J. Trager, Anthony N. Baumann, Romeo‐Paolo T. Perfecto, Christine M. Goertz

**Affiliations:** ^1^ Connor Whole Health University Hospitals Cleveland Medical Center Cleveland Ohio USA; ^2^ Department of Family Medicine and Community Health Case Western Reserve University School of Medicine Cleveland Ohio USA; ^3^ Department of Biostatistics and Bioinformatics Clinical Research Training Program Duke University School of Medicine Durham North Carolina USA; ^4^ Department of Orthopaedic Surgery Duke University School of Medicine Durham North Carolina USA; ^5^ Department of Rehabilitation University Hospitals Cleveland Medical Center Cleveland Ohio USA; ^6^ College of Medicine Northeast Ohio Medical University Rootstown Ohio USA; ^7^ Department of Whole Health Roseburg VA Health Care System Roseburg Oregon USA; ^8^ Duke Clinical Research Institute Duke University School of Medicine Durham North Carolina USA; ^9^ Robert J. Margolis, MD, Center for Health Policy Duke University Durham North Carolina USA

## Abstract

**Background:**

Cauda equina syndrome is a surgical emergency often caused by lumbar disc herniation. Spinal manipulative therapy is commonly used for lumbar spine disorders, but case reports have raised concerns it may precipitate cauda equina syndrome. One cohort study suggested no increased risk, although it did not focus on patients with lumbar spine disorders pertinent to cauda equina syndrome, such as disc herniation, stenosis, or radiculopathy/sciatica.

**Objective:**

To address this evidence gap, we tested the null hypothesis that there is no increased risk of cauda equina syndrome following spinal manipulative therapy among adults with these lumbar spine disorders compared to matched controls receiving physical therapist–led therapeutic exercise (PTE).

**Methods:**

Using a retrospective cohort design, we queried a U.S. research network (TriNetX) including patients aged ≥18 years with a lumbar spine disorder and excluding those with preexisting cauda equina syndrome, incontinence, serious spinal pathology, and recent spine surgery/injection. Data spanned 2005–2025. Patients were divided into cohorts: (1) spinal manipulative therapy administered by a chiropractor or (2) PTE without spinal manipulative therapy. Propensity score matching controlled for confounding variables. Outcomes included the risk ratio of cauda equina syndrome (primary), and bladder catheterization and bowel incontinence as additional cauda equina syndrome markers (secondary).

**Results:**

After matching, there were 34,376 patients in each cohort. Comparing the spinal manipulative therapy cohort to PTE cohort, the incidence and risk of cauda equina syndrome did not significantly differ (risk ratio=0.88 [95% CI, 0.43–1.79]; *p* = .715). The risk of bladder catheterization (risk ratio = 0.50 [95% CI, 0.39– 0.64]; *p* < .001) and fecal incontinence (risk ratio = 0.50 [0.37, 0.68]; *p* < .001) was significantly lower in the spinal manipulative therapy cohort.

**Conclusion:**

Among adults with lumbar disc herniation, stenosis, and/or radiculopathy, we did not identify an association between spinal manipulative therapy and an increased risk of cauda equina syndrome.

## INTRODUCTION

Cauda equina syndrome (CES) is a clinically defined syndrome characterized by at least one of the following: bladder or bowel dysfunction, reduced sensation in the saddle area, or sexual dysfunction.^1^ It is a surgical emergency typically caused by a lumbar disc herniation compressing the lumbosacral nerve roots.^2^ Lumbar stenosis may also cause CES, especially as narrowing of the spinal canal lowers the threshold for nerve compression with smaller disc herniations.^3^, ^4^ Low back pain and sciatica (ie, radiculopathy) are common symptoms of CES, each present in >90% of patients.^5^ CES generally affects adults, with a mean age of 50 years,^4^ and has an incidence of 270 per 100,000 (0.27%) per year among those with low back pain.^6^

Patients with early signs of CES occasionally present to direct‐access clinicians (ie, those whom patients can see without a referral) who manage low back pain, such as primary care physicians,^5^, ^7^ chiropractors,^8^, ^9^, ^10^ and physical therapists.^11^ Because chiropractors frequently treat patients with low back pain or radiculopathy^12^ by using hands‐on manipulation of the spinal joints (ie, spinal manipulation therapy [SMT]), concern has been raised regarding an association between SMT and CES. Chiropractors are trained to recognize the clinical features of CES and refer suspected patients for emergency surgical care.^13^ However, CES may be preceded by mild initial symptoms such as low back pain for a median of 30 days.^14^, ^15^ Additionally, only 27% of patients with CES initially present with more overt symptoms of loss of bowel or bladder function,^16^ making this condition challenging to recognize even among trained clinicians. This dilemma underscores the need to investigate whether SMT could precipitate CES in patients with predisposing lumbar spine disorders who may have subtle CES‐related symptoms.

Previous case reports and medicolegal cases have described CES after SMT, raising concerns that SMT could trigger CES in predisposed patients.^17^, ^18^ Despite this, there is a paucity of high‐level evidence examining the potential SMT–CES association.^19^ Observational studies and randomized controlled trials focusing on patients with lumbar disc herniation and/or radiculopathy have supported the safe use of SMT for sciatica without causing CES.^20^, ^21^, ^22^, ^23^ However, trials may be underpowered to examine this potential association and may include patients with less severe or less acute symptoms than those encountered in real‐world clinical practice.^20^, ^21^, ^22^, ^23^ Chiropractors are often the first clinicians to see patients with disc herniation, radiculopathy, or stenosis,^12^ necessitating additional research focused on this population.

Our recent retrospective cohort study including 134,440 propensity‐matched U.S. adults with low back pain found no significant increased risk of CES following SMT compared to those undergoing an examination by a physical therapist.^19^ Although these findings are reassuring, they may not generalize to patients at greater risk of CES. Specifically, in our previous study only ∼1% of patients had lumbar disc herniation with radiculopathy at baseline, 4% had sciatica, and 3% had lumbar stenosis.^19^ Accordingly, the present study aims to build upon these findings by focusing exclusively on a population predisposed to CES, requiring all patients have either lumbar disc herniation, stenosis, or sciatica/radiculopathy at baseline. This study also builds on the prior methods by (1) requiring physical therapist treatment (ie, exercise) rather than evaluation alone to reduce bias from potential baseline CES suspicion; (2) examining additional CES‐related outcomes of bladder catheterization and fecal incontinence; (3) applying a broader set of exclusions (eg, rare CES etiologies, functional neurological disorders, recent spine surgery) to improve cohort homogeneity; and (4) incorporating over a year of updated data.

Given the limited research on the topic to date, we aimed to investigate the association between SMT and CES in adults with low back disorders including disc herniation, stenosis, and/or radiculopathy. In this population, we tested the null hypothesis that SMT is not associated with a statistically significant positive risk ratio (RR) of CES compared to matched controls receiving physical therapist‐led therapeutic exercise (PTE), over 3 months follow‐up. Secondary outcomes explored the cumulative incidence of CES, and, because bowel and bladder dysfunction are commonly associated with CES, we also calculated the RRs for bladder catheterization and bowel incontinence.

## MATERIALS AND METHODS

### Study design

The present study used data from TriNetX (TriNetX, LLC, Cambridge, MA, USA), a federated research network platform that provides access to aggregated electronic health record data from a network of 103 large health care organizations covering >141 million patients at the time of our query. With a database query date of January 13, 2025, we included patients who met eligibility criteria anywhere from January 13, 2025, to October 13, 2025 (spanning 20 years to 3 months prior to the query date to allow for adequate follow‐up). This data range was selected to maximize sample size while considering database limitations. Specifically, TriNetX limits analyses to patients meeting eligibility criteria a maximum of 20 years retrospectively.

The platform allows users to conduct queries to analyze patient cohorts, using standardized nomenclatures such as the *International Classification of Diseases, Tenth Revision* (ICD‐10).^24^, ^25^ These codes are automatically interconverted to *Ninth Revision* (ICD‐9) as needed. TriNetX conducts data quality procedures to ensure cleanliness, consistency, correctness, and completeness of data.^25^ All data available via the platform are deidentified in accordance with Health Insurance Portability and Accountability Act guidelines. A visual representation of the study design is shown in Supplemental File 1 Figure S1. Study methods adhere to a registered protocol,^26^ and reporting follows Strengthening the Reporting of Observational Studies in Epidemiology.^27^

The first author's institutional review board (IRB) considers studies using deidentified data from the online TriNetX platform Not Human Subjects Research thereby exempting the present study from institutional review board review and waiving the need for consent.

A key feature of this study is the use of PTE as an active comparator.^28^ Further distinguishing this study from the previous one focused on CES risk after SMT,^19^ the present study explicitly requires patients in the comparator PTE cohort to undergo therapeutic exercise, such that both cohorts receive active care. This design feature aims to make the cohorts more comparable with respect to their complexity and care eligibility. Like chiropractors, physical therapists are also direct‐access clinicians who commonly manage low back pain.^29^ Between 36% and 40% of patients with lumbar disc herniation visit a physical therapist^30^, ^31^ and 16% receive SMT administered by a chiropractor,^31^ suggesting that both cohorts reflect common care pathways for low back disorders. Patients who visit physical therapists for spinal pain are generally more comparable to chiropractic patients than those visiting medical physicians or specialists with respect to socioeconomic factors, insurance coverage, comorbidity burden, and self‐rated health status.^32^ Several commonly used therapeutic exercises such as general and motor control exercises have evidence supporting their use for lumbar disc herniation.^33^, ^34^ Finally, PTE is not associated with an increased risk of CES to our knowledge.^33^

### Participants

#### Eligibility criteria

We included adults at least 18 years old presenting with a low back disorder of lumbar disc herniation, sciatica/radiculopathy, and/or lumbar stenosis (Supplemental File 1 Table S1).

Patients were divided into two cohorts based on either receiving chiropractic SMT (Current Procedural Terminology [CPT]: 98940, 98941, or 98942) or PTE (CPT codes: 97001, 97161, 97162, or 97163 [physical therapy evaluations] and 97110 [therapy procedure using exercise]). Patients were identified at the first co‐occurrence of an eligible lumbar spine disorder (ie, lumbar disc herniation, sciatica/radiculopathy, or stenosis) and SMT or PTE, which served as the index date. This helped avoid prevalent users of SMT or PTE for these disorders for whom CES screening and/or decision‐making could be less impactful on care delivery.

To help ensure data completeness, we required patients to have a previous health care visit between 1 day and 3 years preceding the index date (ie, the date when patients received SMT or PTE and were included in the study). To minimize loss to follow‐up, we required at least one health care visit between 1 day and 3 months of follow‐up.

In addition to the requirement of having a CPT code for SMT administered by a chiropractor, patients receiving SMT were required to have the presence of a segmental dysfunction code for the thoracic or lumbopelvic regions (ie, M99.02, M99.03, M99.04, M99.05) indicating that SMT was applied to any of these regions.^18^ Consequently, patients receiving only cervical SMT were not included.

We excluded patients with preexisting CES, cauda equina injury, spinal cord injury, congenital abnormalities of the cauda equina, conus medullaris syndrome, urinary or fecal incontinence, bladder catheterization,^15^ serious lumbar spinal pathology (ie, fracture, malignancy, infection, and bleeding disorders) that may cause CES,^1^, ^3^ recent spine surgery or injection,^35^, ^36^ those with functional neurological disorders or simulated illness that may mimic CES, and individuals with rare etiologies of CES (eg, nervous system Zoster, Guillain–Barré syndrome)^37^, ^38^ (Table S2). These exclusions aimed to make the cohorts more homogeneous with respect to their low back conditions. Those receiving manual therapy (CPT: 97140), chiropractic SMT (CPT: 98940, 98941, 98942), or osteopathic manipulation (CPT: 1013558) were excluded from the PTE cohort. This strategy aimed to minimize exposure misclassification considering some physical therapists may provide SMT,^39^ or patients could receive SMT concurrently from osteopaths or chiropractors surrounding the index date. Exclusions are detailed in Supplemental File 1 Table S2.

### Variables

We used propensity score matching to minimize bias by balancing variables associated with CES,^28^ including age, gender, comorbidities, degenerative lumbar spine conditions that may cause CES (eg, disc herniation, spondylolisthesis, stenosis), other CES risk factors such as trauma and neurological disorders, and medications used for low back pain that increase the likelihood of urinary retention.^37^, ^38^, ^40^, ^41^, ^42^ Variables available within 1 year preceding the index date were eligible for matching (Supplemental File 1 Table S3).

### Primary outcome

We ascertained new instances of CES (ICD‐10: G83.4) over a 3‐month window including and following the index date. Previous studies established that although the median time to diagnose CES is <2 weeks, patients may have symptoms up to 3 months or more before being diagnosed.^43^, ^44^, ^45^, ^46^ Shorter time windows (eg, 0 and 1 day) were avoided as these may miss a substantial number of CES diagnoses. Longer windows were avoided considering this would allow for additional confounding due to other intervening factors after the index date (eg, discontinuation of treatment or use of other therapies). We presented findings in the unmatched sample as a form of sensitivity analysis.

### Secondary outcomes

We analyzed postmatching secondary outcomes apart from CES considering that clinicians may append the diagnosis using variable thresholds.^2^, ^5^, ^47^ Accordingly, secondary outcomes were used to determine the robustness of our primary outcome. We examine the RR for new instances of sequelae of CES including urinary catheterization and fecal incontinence.^5^

We further characterized the SMT cohort according to the mean number of follow‐up SMT visits (CPT: 98940, 98941, or 98942). We reported and compared the proportion of patients receiving physical therapy‐related intervention(s) or evaluation(s) between cohorts and reported the number of follow‐up physical therapy visits among patients with nonzero counts of these visits using the mean, SD, and median visit count. A range of codes were used to capture these encounters (Supplemental File 1 Table S4).

### Statistical methods

We conducted statistical analysis using features within the TriNetX database analytics platform. To compare the baseline characteristics, we used standardized mean deviation (SMD) with a threshold of >0.1 indicating between‐cohort imbalance. Propensity score matching was performed using a greedy nearest‐neighbor approach with a 1:1 ratio and a caliper of 0.1 pooled standard deviations. A logistic regression was applied using Python (scikit‐learn version 1.3 [Python Software Foundation, Delaware, USA]) to pooled covariate matrices to predict the probability of each patient being in the control cohort. The matching process cycles through each patient in the smaller cohort and identifies the closest unmatched patient from the larger cohort, excluding non‐matching patients.

We used R (build 4.3.2, Vienna, Austria^48^) to calculate RRs with 95% confidence intervals (CIs), calculate risk difference (RD) for CES and its 95% CI using the Miettinen and Nurminen method, using DescTools,^49^ and derived *p* values for RRs and risk difference using the chi‐square test with a significance threshold of *p* < .05. We also used ggplot2^50^ to plot CES incidence and cumulative incidence per cohort.

We took additional steps to explore data quality and balance diagnostics. This included reporting measures of cohorts' follow‐up duration, the proportion of unknown variables per cohort, and plots of covariate balance and propensity score density using ggplot2.^50^ We explored RRs for negative control outcomes unrelated to SMT^51^, ^52^ (Supplemental File 1 Table S5). These were evaluated by assessing whether the majority of these RRs fell within a predefined range surrounding the null (0.73 ≥ RR ≤1.38), suggestive of between‐cohort balance.^52^, ^53^ Imbalance would suggest that additional matching may be necessary or that caution is needed when interpreting results.

### Study size

We estimated a total required sample of 51,856 using G*Power (Kiel University, DE) z‐tests to examine a difference in incidence proportion between cohorts of 0.015% vs. 0.030%,^6^ using a two‐tailed α‐error of .05, power of 0.95, and allocation ratio of one. Initial test queries suggested this sample size was attainable.

## RESULTS

### Participants

Before matching, there were 34,434 patients in the SMT cohort and 630,160 in the PTE cohort. Compared to the PTE cohort, patients in the SMT cohort were initially younger, with a lower proportion who had been prescribed opioids, benzodiazepines, or gabapentinoids and lower incidence of external causes of morbidity (eg, motor vehicle collisions), among other differences (SMDs >0.1; Table 1). After matching, there were 34,376 patients in each cohort and all variables were adequately matched, having SMD values <0.1. A patient selection flowchart is shown in Figure S2.

**TABLE 1:** Baseline characteristics before and after matching.

Variable; *n* (%)	Before matching			After matching		
	SMT	PTE	SMD	SMT	PTE	SMD
*n*	34,434	630,160	NA	34,376	34,376	NA
Age at index, y	55.8 (15.6)	60.5 (14.8)	0.312	55.8 (15.6)	55.8 (15.9)	0.001
Female	20,352 (59%)	380,323 (60%)	0.025	20,331 (59%)	20,321 (59%)	0.001
Male	14,081 (41%)	232,890 (37%)	0.081	14,044 (41%)	13,991 (41%)	0.003
Unknown gender	10 (0%)	16,947 (3%)	0.231	10 (0%)	64 (0%)	0.048
Other and unspecified diseases of spinal cord	173 (1%)	12,679 (2%)	0.136	173 (1%)	174 (1%)	0.000
Benzodiazepines	6628 (19%)	225,769 (36%)	0.378	6625 (19%)	6654 (19%)	0.002
Inflammatory polyneuropathy	31 (0%)	1808 (0%)	0.045	31 (0%)	34 (0%)	0.003
Diabetes mellitus	5007 (15%)	150,446 (24%)	0.239	5007 (15%)	5045 (15%)	0.003
Parkinson's disease	180 (1%)	8655 (1%)	0.088	180 (1%)	172 (0%)	0.003
Benign prostatic hyperplasia	1550 (5%)	39,594 (6%)	0.079	1547 (4%)	1519 (4%)	0.004
Injuries to the thorax	1799 (5%)	25,416 (4%)	0.057	1753 (5%)	1723 (5%)	0.004
Cerebrovascular diseases	1150 (3%)	52,442 (8%)	0.214	1150 (3%)	1124 (3%)	0.004
Foot drop	119 (0%)	6779 (1%)	0.087	119 (0%)	109 (0%)	0.005
Mood disorders	6669 (19%)	151,991 (24%)	0.115	6663 (19%)	6591 (19%)	0.005
Ankylosing spondylitis	51 (0%)	1847 (0%)	0.031	51 (0%)	44 (0%)	0.005
Other paralytic syndromes	21 (0%)	1681 (0%)	0.051	21 (0%)	26 (0%)	0.006
Anxiety disorders	7290 (21%)	154,460 (25%)	0.080	7277 (21%)	7195 (21%)	0.006
Overweight and obesity	6850 (20%)	153,648 (24%)	0.108	6845 (20%)	6756 (20%)	0.006
Peripheral neuropathy	1408 (4%)	52,503 (8%)	0.176	1408 (4%)	1360 (4%)	0.007
Abdominal, lumbar, or pelvic injury	3848 (11%)	41,641 (7%)	0.161	3790 (11%)	3700 (11%)	0.008
External causes of morbidity	1892 (5%)	106,365 (17%)	0.367	1892 (6%)	1962 (6%)	0.009
Opioid analgesics	11,727 (34%)	335,176 (53%)	0.393	11,720 (34%)	11,865 (35%)	0.009
Chronic inflammatory demyelinating polyneuritis	10 (0%)	859 (0%)	0.037	10 (0%)	16 (0%)	0.009
Injury of unspecified body region	611 (2%)	19,903 (3%)	0.089	611 (2%)	569 (2%)	0.009
Demyelinating diseases	185 (1%)	6175 (1%)	0.051	185 (1%)	162 (0%)	0.009
Spondylolisthesis, lumbar region	606 (2%)	28,602 (5%)	0.160	606 (2%)	557 (2%)	0.011
Genitourinary symptoms/signs	3944 (11%)	98,321 (16%)	0.122	3943 (11%)	3818 (11%)	0.011
Postlaminectomy syndrome	256 (1%)	8327 (1%)	0.057	256 (1%)	221 (1%)	0.012
Multiple sclerosis	163 (0%)	5333 (1%)	0.046	163 (0%)	134 (0%)	0.013
Other diseases of the urinary system	2917 (8%)	85,462 (14%)	0.163	2917 (8%)	2793 (8%)	0.013
Tricyclic antidepressants	1497 (4%)	31,643 (5%)	0.032	1496 (4%)	1399 (4%)	0.014
Spinal stenosis, lumbosacral region	270 (1%)	11,294 (2%)	0.089	270 (1%)	227 (1%)	0.015
Fibromyalgia	3025 (9%)	33,715 (5%)	0.134	2975 (9%)	2828 (8%)	0.015
Gabapentinoids	5099 (15%)	181,446 (29%)	0.344	5099 (15%)	4870 (14%)	0.019
Skeletal muscle relaxants	7578 (22%)	196,349 (31%)	0.208	7571 (22%)	7282 (21%)	0.020
Spinal stenosis, lumbar region	2081 (6%)	83,608 (13%)	0.246	2081 (6%)	1863 (5%)	0.027
Other inflammatory spondylopathies	1171 (3%)	24,645 (4%)	0.027	1169 (3%)	979 (3%)	0.032
Chronic pain	7082 (21%)	202,020 (32%)	0.263	7080 (21%)	6549 (19%)	0.039

*Note*: Variables are ordered as follows: number of patients (*n*), demographics; then by increasing standardized mean difference after matching.

Abbreviations: PTE, physical therapist‐led therapeutic exercise; SMD, standardized mean difference; SMT, spinal manipulative therapy.

### Data quality

After matching, the propensity score densities of each cohort were superimposed (Supplemental File 1 Figure S3), and all SMD values were below the threshold for balance (Table 1; Supplemental File 1 Figure S4). The proportion of patients with an unknown gender was approximately 0% in both cohorts (SMD = 0.048). Comparing the SMT to PTE cohort over the 90‐day follow‐up, the RRs for negative control outcomes were in the target zone for balance. This included colonoscopy (RR = 1.28 [95% CI, 1.14–1.44]), azithromycin prescription (RR = 1.13 [95% CI, 1.04–1.23]), and acute upper respiratory infection (RR = 1.21 [95% CI, 1.13–1.30]). The proportion of patients who had data spanning at least the 90‐day follow‐up was high in each cohort (SMT: 96.7%; PTE: 94.5%), and the mean and median follow‐up duration were also similar between cohorts (Supplemental File 1, Figure S5 and Table S6). Accordingly, these findings suggest the presence of adequate and similar markers of data completeness, indicate that propensity matching was successful, and suggest the absence of differential attrition between cohorts.

### Primary outcome

**FIGURE 1:**
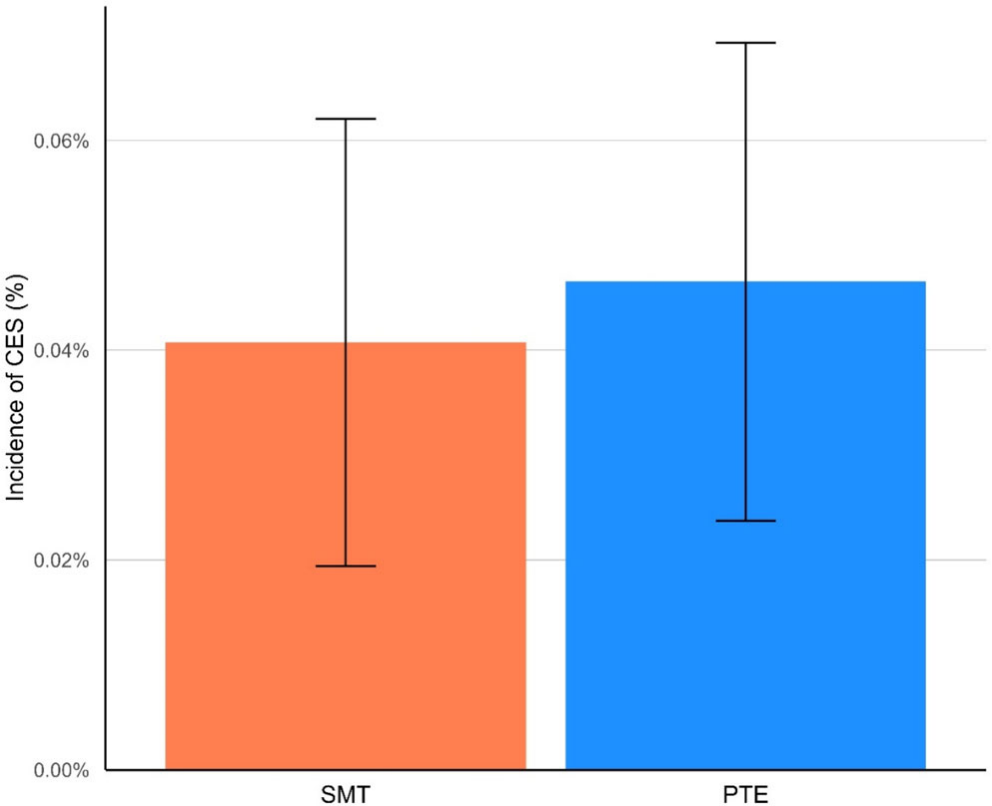
Total incidences of CES per cohort over a 90‐day follow‐up window. The incidence of CES in the cohort receiving spinal manipulative therapy is shown in orange, and the incidence of CES in the cohort receiving physical therapist‐led therapeutic exercise is shown in blue. The error bars demonstrate 95% confidence intervals, which overlap substantially suggesting no meaningful between‐cohort difference. CES, cauda equina syndrome; PTE, physical therapist‐led therapeutic exercise; SMT, spinal manipulative therapy.

**FIGURE 2:**
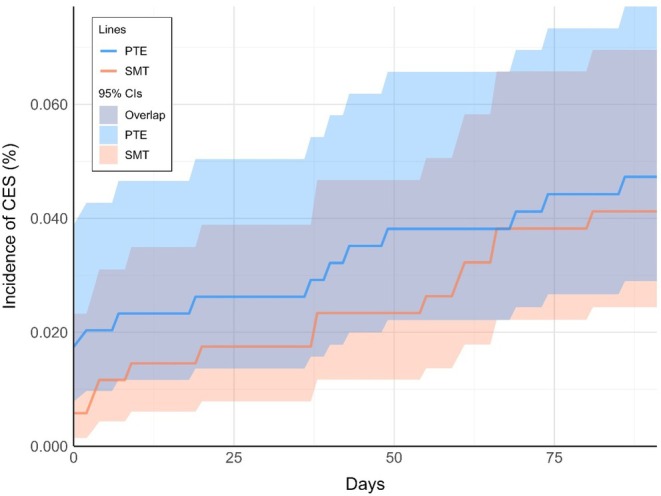
Cumulative incidence of CES per cohort over a 90‐day follow‐up window. The incidence of CES in the cohort receiving spinal manipulative therapy is shown in orange, and the incidence of CES in the cohort receiving physical therapist‐led therapeutic exercise is shown in blue. The shaded regions demonstrate 95% confidence intervals. Substantial overlap between confidence intervals is evident, as well as superimposition of the incidence during the latter half of follow‐up. CES, cauda equina syndrome; CI, confidence interval; PTE, physical therapist‐led therapeutic exercise; SMT, spinal manipulative therapy.

Following matching, the total number of CES cases was 14 in the SMT cohort and 16 in the PTE cohort. Comparing the SMT cohort to PTE cohort, the incidence and risk of CES did not significantly differ (0.04% vs. 0.05%; RR = 0.88 [95% CI, 0.43–1.79]; *p* = .715), as presented in Table 2 and Figures 1 and 2. The RD further suggested no meaningful difference in risk of CES (RD = −0.01% [95% CI, −0.04% to 0.03%]; *p* = .715).

**TABLE 2:** Primary outcome findings.

Measure	Before matching		After matching*	
	SMT	PTE	SMT	PTE
Total patients	34,434	630,160	34,376	34,376
Patients with CES (n)	14	387	14	16
CES n % (95% CI)	0.04% (0.02% to 0.06%)	0.06% (0.06% to 0.07%)	0.04% (0.02% to 0.06%)	0.05% (0.02% to 0.07%)
Risk difference (95% CI; p)	−0.02% (−0.04% to 0.01%; *p* = .127)	NA	−0.01% (−0.04% to 0.03%; *p* = .715)	NA
Risk ratio (95% CI; p)	0.66 (0.39, 1.13; *p* = .127)	NA	0.88 (0.43 to 1.79; *p* = .715)	NA

*Note*: Results for cauda equina syndrome are shown both before and after matching* (primary outcome), comparing the cohorts receiving spinal manipulative therapy and physical therapist‐led therapeutic exercise.

Abbreviations: CES, cauda equina syndrome; CI, confidence interval; NA, not applicable; PTE, physical therapist‐led therapeutic exercise; SMT, spinal manipulative therapy.

### Secondary outcomes

Following matching, comparing the SMT cohort to the PTE cohort, the incidence and risk of bladder catheterization (RR = 0.50 [95% CI, 0.39–0.64]; *p* < .001) and fecal incontinence (RR = 0.50 [95% CI, 0.37–0.68]; *p* < .001) were significantly lower, as presented in Table 3. Total and cumulative incidences for these outcomes are demonstrated in Supplemental File 1 Figures S6– S9.

**TABLE 3:** Secondary outcome findings.

Outcome and measure	SMT	PTE
Total patients	34,376	34,376
Bladder catheterization		
Bladder catheterization *n*	91	183
Bladder catheterization % (95% CI)	0.26% (0.21% to 0.32%)	0.53% (0.46% to 0.61%)
*n* per 100,000 (95% CI)	264.7 (210.4 to 319.0)	532.3 (455.4 to 609.3)
Risk difference (95% CI; *p* value)	−0.27% (−0.36% to −0.17%; *p* < .001)	NA
Risk ratio (95% CI; *p* value)	0.50 (0.39 to 0.64; *p* < .001)	NA
Fecal incontinence		
Fecal incontinence n	64	127
Fecal incontinence % (95% CI)	0.19% (0.14% to 0.23%)	0.37% (0.31% to 0.43%)
*n* per 100,000 (95% CI)	186.2 (140.6 to 231.7)	369.4 (305.3 to 433.6)
Risk difference (95% CI; *p* value)	−0.18% (−0.26% to −0.11%; *p* < .001)	NA
Risk ratio (95% CI; *p* value)	0.50 (0.37 to 0.68; *p* < .001)	NA

*Note*: Results for bladder catheterization and fecal incontinence are shown after matching, comparing the cohorts receiving spinal manipulative therapy and physical therapist‐led therapeutic exercise.

Abbreviations: CI, confidence interval; PTE, physical therapist‐led therapeutic exercise; SMT, spinal manipulative therapy.

Patients receiving SMT received a mean (SD) 6.2 (5.5) sessions of SMT during the 90‐day follow‐up window, yielding an estimated total of 213,131 SMT sessions for the entire cohort. In the SMT cohort, 15,008 patients received physical therapy‐related intervention(s) or evaluation(s), and 22,552 patients in the PTE cohort received these therapies during the 90‐day follow‐up. Accordingly, the incidence and likelihood of receiving any physical therapy intervention or evaluation were significantly lower in the SMT cohort compared to PTE cohort (43.66% vs. 65.60%; RR = 0.67 [95% CI, 0.66–0.68]; *p* < .0001). However, among individuals receiving physical therapy‐related follow‐up visits, the mean count of such visits was slightly greater in the SMT cohort [SD] (mean = 5.7 [5.2]) versus PTE cohort (mean = 5.1 [4.7]) with both cohorts having a median of four visits.

## DISCUSSION

This study found that CES risk does not increase after SMT compared to PTE among adults with lumbar disc herniation, radiculopathy, or stenosis. Our use of propensity score matching helps ensure that these findings were independent of recognized confounders. The cumulative incidence data further support that CES occurs at similar rates in the study population, regardless of treatment received being SMT or PTE. Our secondary outcomes corroborate these results, showing no significant increase in CES‐related events such as bladder catheterization or bowel incontinence among SMT recipients. Additionally, our data suggest that PTE is relatively safe considering the low overall incidence of CES, which was not significantly different between cohorts after matching.

Direct comparisons of our observed CES incidence with previous incidence estimates are precluded by differences in study populations and methods. However, there are similarities in the present CES incidence with the general low back pain population, rather than a potential increase related to our focus on disc herniation, radiculopathy, and stenosis. The incidence estimates for CES in our study, which range from 0.04% to 0.06% across both cohorts, align with a prior study indicating that CES affects 270 per 100,000 (0.27% [95% CI, 0.14%–0.54%]) annually among adults with low back pain presenting to secondary care settings,^6^ translating to an incidence of approximately 0.07% per 90 days.

One explanation of the similar CES incidence values between our study, which focused on patients with lumbar spine disorders, and prior studies focusing on a more general low back pain population may relate to our exclusion of patients with other neurological disorders. These exclusions likely reduced our observed CES incidence by avoiding ascertainment of scan‐negative CES.^5^, ^37^, ^38^ Another explanation for the similar incidence is that patients with more severe initial presentations of lumbar spine disorders could have avoided seeking SMT or PTE and instead presented to an emergency setting^6^ and were not included in our study. Similarly, any patients for whom chiropractors or physical therapists suspected CES and referred for emergency surgical evaluation rather than providing treatment have been excluded, thereby counterbalancing any potential increase in observed CES incidence in this population.

Our eligibility criteria aimed to reflect real‐world chiropractic practice by excluding patients with serious spinal pathologies who would not typically see a chiropractor and for whom SMT is contraindicated (eg, those with recent spine fracture, spinal infection, and known CES‐related conditions).^54^, ^55^, ^56^, ^57^ This selective narrowing likely excluded patients with high CES risk. However, our design focused on patients with lumbar disc herniation, sciatica/radiculopathy, and/or stenosis, which represent the primary etiologies or risk factors for CES.^2^, ^3^, ^4^, ^5^ Chiropractors regularly use SMT for these lumbar spine disorders in the absence of overt CES features.^12^ Accordingly, our study design allowed us to test the potential risk association of SMT in a clinically relevant population.

The findings suggesting a significantly reduced likelihood of bladder catheterization and fecal incontinence among SMT recipients should be interpreted with caution. These outcomes are not specific to CES and may arise from various benign reasons. Chiefly, patients receiving PTE may have had undiagnosed or concurrent genitourinary or bowel dysfunction at baseline. For instance, individuals with interstitial cystitis may experience pelvic floor dysfunction and seek physical therapy, while also undergoing bladder instillation, a procedure that requires catheterization.^58^ Propensity score matching cannot feasibly control for all variables influencing bladder or bowel function, as doing so would lead to excessive trimming and potentially widen the effect estimate precision for our main outcome of CES.^59^ Our findings do not imply that PTE contributes to bladder catheterization or fecal incontinence, and instead should be viewed as reinforcing our primary null hypothesis.

The findings of our study are supported by various experimental and observational studies. For instance, randomized clinical trials^21^, ^22^ and multiple prospective observational studies^20^, ^60^, ^61^ investigating SMT for lumbar disc herniation or radiculopathy reported no serious adverse events such as CES. One chart review that examined potential adverse events after 960,140 chiropractic SMT sessions identified no cases of CES.^62^ Additionally, one retrospective study identified a reduction in the likelihood of discectomy among SMT recipients compared to matched controls, serving as a proxy for the safety of SMT considering CES typically necessitates surgical intervention.^63^ Although biomechanical data on this topic remain limited, one observational study found no significant short‐term changes in disc herniation size or morphology following SMT,^64^ suggesting that SMT likely does not acutely worsen disc herniations.

Our study helps contextualize the medicolegal and case reports documenting CES following spinal SMT,^17^, ^18^ suggesting caution in their interpretation due to inherent publication bias and the absence of epidemiological methods or control groups. SMT and PTE are common entry points for lumbar spine disorders,^29^, ^30^, ^31^, ^65^ and CES may occur more often in this population regardless of treatments rendered. Accordingly, SMT or PTE may have been incidentally performed while CES was developing, as may initially present with early or benign‐appearing symptoms.^16^

Another explanation for the observed absence of increased risk of CES after SMT is that chiropractors may tailor SMT techniques to the specific lumbar spine disorder(s) present (ie, lumbar disc herniation, radiculopathy, and/or stenosis). A range of SMT techniques is available, spanning from low to high force, and including both thrust and nonthrust varieties. One example is flexion‐distraction, a technique involving nonthrust mobilization and manual traction. A survey of 280 chiropractors indicated that although only 29% of respondents reported using flexion‐distraction for lumbar disc herniation with radiculopathy, this technique was used more frequently for this condition compared to other diagnoses, such as facet syndrome and myofascial pain.^66^ However, thrust manipulation was still commonly used in these patients, with approximately 53% of respondents indicating they would use it in such cases.^66^ Regardless, the established variety of approaches, combined with the lack of observed risk for CES, may indicate proficiency in managing lumbar spine disorders. Nevertheless, it remains essential for chiropractors to conduct thorough examinations and remain vigilant in assessing patients for CES, ensuring timely referral for emergency surgical evaluation when necessary.^13^, ^56^

Existing care guidelines recommend SMT either as a stand‐alone intervention^67^ or as part of a broader multimodal strategy^68^, ^69^, ^70^, ^71^, ^72^ for patients with lumbar disc herniation and/or radiculopathy. The evidence supports the relative safety of SMT within this population, suggesting that SMT can be a viable treatment option in the absence of clinical features of CES or progressive motor loss.^73^, ^74^ Although clinical guidelines have found insufficient evidence for use of SMT for lumbar stenosis,^74^ this condition appears to have made up only a small proportion of the current study population. Recent evidence regarding the utility of SMT in lumbar stenosis has been promising,^75^ although additional studies devoted to the safety and effectiveness of SMT are needed.

Questions remain regarding the mechanisms underlying the observed null SMT–CES association. For instance, the present study does not elucidate whether our findings reflect the inherent safety of SMT itself, the clinical decision‐making processes of chiropractors, or a combination of both. Future research including more granular data extracted from clinical charts could examine how chiropractors and physical therapists manage patients with lumbar disc herniation, radiculopathy, and stenosis. Such studies could investigate examination thoroughness and diagnosis and referral patterns of CES, potential associations between specific SMT techniques and CES, or patient selection criteria when determining whether to administer SMT or exercise. Additional surveys exploring how chiropractors might modify treatments for patients with lumbar disc herniation would also be valuable.^66^ Finally, given that CES is rare but serious, developing prediction models to identify which patients presenting for SMT or PTE may be at highest risk could facilitate earlier triage and surgical evaluation. Such studies could incorporate regression models with predictors including demographics, comorbidities, body mass index, and clinical variables such as pain severity, disability, or specific examination findings.

## STRENGTHS AND LIMITATIONS

The present study strengths include an interdisciplinary investigator team, a priori protocol,^26^ and methodological improvements compared to the previous study on this topic including^19^ (1) focus on a population with a greater baseline risk of CES, (2) use of a more active comparator undergoing PTE, (3) a broader set of exclusions, (4) more comprehensive propensity matching strategy, (5) use of outcome negative controls, and (6) addition of secondary outcomes.

Several limitations are noteworthy. The relatively low number of observed CES events per cohort, yielding wide 95% CIs for incidence estimates, reflects the rarity of CES and limits precision of our effect estimates. This warrants some caution in interpretation, even though the study was adequately powered via a priori calculation, with >68,000 patients, and corroborates a previous study.^19^ A follow‐up study with a larger sample size could be conducted using the TriNetX Linked Network, which links electronic health records with claims data, including >1.5 million chiropractic patients (vs. ~150,000 in the current Research Network).^24^, ^76^, ^77^ A key factor leading to our observed imprecision is the comparatively small chiropractic cohort size, whereas the larger PTE cohort produced narrower incidence estimates prior to matching. A follow‐up study using TriNetX Linked would require some adaptation of the present methods, for instance a narrowing of the data range to match the range in Linked (approximately 15 years). Overall, this could yield up to a 10‐times larger chiropractic cohort size, improved precision, and potentially greater generalizability to broader private practice chiropractic settings.

There is a lack of definitive diagnostic criteria for CES, and there is a potential for scan‐negative CES.^5^, ^37^, ^38^ Although the potential impact of false‐positive CES diagnosis is mitigated by our selection criteria and matching strategy, an artificial increase in CES incidence in one or both cohorts remains possible. Regardless, we have no reason to suspect that documentation bias would differ between cohorts, which were both engaged in large health care organizations. We are unable to compare our query against chart review given the deidentified nature of the dataset. There may be unmeasured confounding related to between‐cohort differences related to patients' baseline pain severity, disability level, or symptom distribution due to limitations in ICD‐10 granularity. Furthermore, we were unable to determine biomechanical or technique parameters of SMT used, such as the type of thrust, patient positioning, or force‐time characteristics, due to limited data granularity and an inability to observe patient‐level information. In addition, we could not determine whether SMT procedures were modified in response to comorbidities or precautions such as lumbar stenosis or osteoporosis.^62^, ^66^ We were also unable to determine the specific types of therapeutic exercises administered in the PTE cohort. Finally, the findings may not generalize to other countries wherein the incidence and treatment pathways for CES may vary.

## CONCLUSION

The present study identified no increase in risk of CES or secondary markers of this condition among adults with lumbar spine disorders receiving SMT compared to matched controls receiving PTE. These findings suggest that the likelihood of CES following chiropractor‐administered SMT is similar to that following exercise administered by a physical therapist in this population. Based on an adequately powered sample of 68,752 patients, these results are reassuring and corroborate previous research on the topic. Replication using a larger dataset could enhance the precision of CES incidence estimates and improve the certainty of findings.

## FUNDING INFORMATION

This project is supported by the Clinical and Translational Science Collaborative of Northern Ohio which is funded by the National Institutes of Health, National Center for Advancing Translational Sciences, Clinical and Translational Science Award grant, UM1TR004528. The work conducted by R.T. received support from the Elisabeth Severance Prentiss Foundation (Cleveland, OH) through general funding. The study did not receive a specific grant, and the funders were not involved in developing the study protocol or any key decisions.

## ETHICS STATEMENT

The University Hospitals Institutional Review Board (Cleveland, OH, USA) considers studies using deidentified data from the online TriNetX platform to be Not Human Subjects Research thereby exempting the present study from institutional review board review and waiving the need for consent.

## DISCLOSURE

Robert J. Trager acknowledges that he has received royalties as the author of two texts on the topic of sciatica. The other authors have declared no competing interests.

This journal‐based CME activity is designated for 1.0 *AMA PRA Category 1 Credit* ^TM^. Effective January 2024, learners are no longer required to correctly answer a multiple‐choice question to receive CME credit. Completion of an evaluation is required, which can be accessed using this link, https://onlinelearning.aapmr.org/. This activity is FREE to AAPM&R members and available to nonmembers for a nominal fee. CME is available for 3 years after publication date. For assistance with claiming CME for this activity, please contact (847) 737–6000. All financial disclosures and CME information related to this article can be found on the Online Learning Portal (https://onlinelearning.aapmr.org/) prior to accessing the activity.

## Supporting information


**Data S1.** Supporting Information.

## Data Availability

Minimal, deidentified, aggregate data for our primary outcome, cumulative incidence, and propensity score densities are available in figshare (https://doi.org/10.6084/m9.figshare.28234589).

## References

[1] Fraser S , Roberts L , Murphy E . Cauda equina syndrome: a literature review of its definition and clinical presentation. Arch Phys Med Rehabil. 2009;90:1964‐1968. 19887225 10.1016/j.apmr.2009.03.021

[2] Mustafa MA , Richardson GE , Gillespie CS , et al. Definition and surgical timing in cauda equina syndrome–an updated systematic review. PLoS One. 2023;18:e0285006. doi:10.1371/journal.pone.0285006 37141301 PMC10159340

[3] Kuris EO , McDonald CL , Palumbo MA , Daniels AH . Evaluation and management of cauda equina syndrome. Am J Med. 2021;134:1483‐1489. doi:10.1016/j.amjmed.2021.07.021 34473966

[4] Thakur JD , Storey C , Kalakoti P , et al. Early intervention in cauda equina syndrome associated with better outcomes: a myth or reality? Insights from the Nationwide Inpatient Sample database (2005–2011). Spine J. 2017;17:1435‐1448. 28456676 10.1016/j.spinee.2017.04.023

[5] Woodfield J , Hoeritzauer I , Jamjoom AAB , et al. Presentation, management, and outcomes of cauda equina syndrome up to one year after surgery, using clinician and participant reporting: a multi‐centre prospective cohort study. Lancet Reg Health Eur. 2023;24:100545. doi:10.1016/j.lanepe.2022.100545 36426378 PMC9678980

[6] Hoeritzauer I , Wood M , Copley PC , Demetriades AK , Woodfield J . What is the incidence of cauda equina syndrome? A systematic review. J Neurosurg Spine. 2020;32:1‐841. doi:10.3171/2019.12.SPINE19839 32059184

[7] Henschke N , Maher CG , Refshauge KM , et al. Prevalence of and screening for serious spinal pathology in patients presenting to primary care settings with acute low back pain. Arthritis Rheum. 2009;60:3072‐3080. doi:10.1002/art.24853 19790051

[8] Bodalia RT , Bogar WC , Rivera‐Melo H . Cauda equina syndrome following lumbar disc herniation at L5‐S1: a case report. J Chiropr Med. 2021;20:158‐162. doi:10.1016/j.jcm.2021.12.007 35463844 PMC9023130

[9] Elderfield G . Cauda equina syndromes in conservative care: four case reports. Br J Chiropr. 1999;3:98‐102. doi:10.1016/S1466-2108(99)90053-0

[10] Busse JW , Hsu WS . Rapid progression of acute sciatica to cauda equina syndrome. J Manip Physiol Ther. 2001;24:350‐355. doi:10.1067/mmt.2001.115261 11416826

[11] Budtz CR , Hansen RP , Thomsen JNL , Christiansen DH . The prevalence of serious pathology in musculoskeletal physiotherapy patients – a nationwide register‐based cohort study. Physiotherapy. 2021;112:96‐102. doi:10.1016/j.physio.2021.03.004 34034209

[12] Himelfarb I , Hyland J , Ouzts N , et al. National Board of Chiropractic Examiners: Practice Analysis of Chiropractic 2020. NBCE; 2020.

[13] Souza T . Lumbopelvic Complaints. Differential Diagnosis and Management for the Chiropractor: Protocols and Algorithms. Jones & Bartlett Learning; 2008:143‐201.

[14] Gardner A , Gardner E , Morley T . Cauda equina syndrome: a review of the current clinical and medico‐legal position. Eur Spine J. 2011;20:690‐697. 21193933 10.1007/s00586-010-1668-3 PMC3082683

[15] Korse NS , Pijpers JA , van Zwet E , Elzevier HW , Vleggeert‐Lankamp CLA . Cauda equina syndrome: presentation, outcome, and predictors with focus on micturition, defecation, and sexual dysfunction. Eur Spine J. 2017;26:894‐904. doi:10.1007/s00586-017-4943-8 28102451

[16] Daniels EW , Gordon Z , French K , Ahn UM , Ahn NU . Review of medicolegal cases for cauda equina syndrome: what factors lead to an adverse outcome for the provider? Orthopedics. 2012;35:e414‐e419. doi:10.3928/01477447-20120222-15 22385455

[17] Hebert JJ , Stomski NJ , French SD , Rubinstein SM . Serious adverse events and spinal manipulative therapy of the low back region: a systematic review of cases. J Manip Physiol Ther. 2015;38:677‐691. 10.1016/j.jmpt.2013.05.009 23787298

[18] Boucher P , Robidoux S . Lumbar disc herniation and cauda equina syndrome following spinal manipulative therapy: a review of six court decisions in Canada. J Forensic Leg Med. 2014;22:159‐169. doi:10.1016/j.jflm.2013.12.026 24485443

[19] Trager RJ , Baumann AN , Perez JA , Dusek JA , Perfecto R‐PT , Goertz CM . Association between chiropractic spinal manipulation and cauda equina syndrome in adults with low back pain: retrospective cohort study of US academic health centers. PLoS One. 2024;19:e0299159. doi:10.1371/journal.pone.0299159 38466710 PMC10927125

[20] McMorland G , Suter E , Casha S , du Plessis SJ , Hurlbert RJ . Manipulation or microdiskectomy for sciatica? A prospective randomized clinical study. J Manip Physiol Ther. 2010;33:576‐584. doi:10.1016/j.jmpt.2010.08.013 21036279

[21] Ghasabmahaleh SH , Rezasoltani Z , Dadarkhah A , Hamidipanah S , Mofrad RK , Najafi S . Spinal manipulation for subacute and chronic lumbar radiculopathy: a randomized controlled trial [with consumer summary]. Am J Med. 2021;134(1):135‐141. 32931763 10.1016/j.amjmed.2020.08.005

[22] Santilli V , Beghi E , Finucci S . Chiropractic manipulation in the treatment of acute back pain and sciatica with disc protrusion: a randomized double‐blind clinical trial of active and simulated spinal manipulations. Spine J. 2006;6(2):131‐137. 16517383 10.1016/j.spinee.2005.08.001

[23] Singh V , Malik M . Efficacy of manual therapy interventions in management of lumbar prolapsed intervertebral disc: a pilot randomized controlled trial. Ro J Neurol. 2021;20:373‐378.

[24] Palchuk MB , London JW , Perez‐Rey D , et al. A global federated real‐world data and analytics platform for research. JAMIA Open. 2023;6:ooad035. doi:10.1093/jamiaopen/ooad035 37193038 PMC10182857

[25] Topaloglu U , Palchuk MB . Using a federated network of real‐world data to optimize clinical trials operations. JCO Clin Cancer Inform. 2018;2:1‐10. doi:10.1200/CCI.17.00067 PMC6816049 30652541

[26] Trager RJ , Baumann A , Perfecto R‐PT , Goertz C . Among adults with disc herniation, stenosis, and radiculopathy, is spinal manipulation associated with increased risk of cauda equina syndrome? A cohort study protocol. 2025. doi:10.17605/OSF.IO/FQX6Z

[27] von Elm E , Altman DG , Egger M , Pocock SJ , Gøtzsche PC , Vandenbroucke JP . The Strengthening the Reporting of Observational Studies in Epidemiology (STROBE) statement: guidelines for reporting observational studies. Ann Intern Med. 2007;147:573‐577. doi:10.2471/blt.07.045120 17938396

[28] Gokhale M , Stürmer T , Buse JB . Real‐world evidence: the devil is in the detail. Diabetologia. 2020;63:1694‐1705. doi:10.1007/s00125-020-05217-1 32666226 PMC7448554

[29] Bise CG , Schneider M , Freburger J , et al. First provider seen for an acute episode of low Back pain influences subsequent health care utilization. Phys Ther. 2023;103:pzad067. doi:10.1093/ptj/pzad067 37379349

[30] Thackeray A , Fritz JM , Lurie JD , Zhao W , Weinstein JN . Nonsurgical treatment choices by individuals with lumbar intervertebral disc herniation in the United States: associations with long‐term outcomes. Am J Phys Med Rehabil. 2017;96:557‐564. doi:10.1097/PHM.0000000000000685 28045705 PMC5494009

[31] Lilly DT , Davison MA , Eldridge CM , et al. An assessment of nonoperative management strategies in a herniated lumbar disc population: successes versus failures. Glob Spine J. 2020;11:2192568220936217. 10.1177/2192568220936217 PMC8351061 32677528

[32] Chevan J , Riddle DL . Factors associated with care seeking from physicians, physical therapists, or chiropractors by persons with spinal pain: a population‐based study. J Orthop Sports Phys Ther. 2011;41:467‐476. doi:10.2519/jospt.2011.3637 21654096

[33] Pourahmadi MR , Taghipour M , Takamjani IE , Sanjari MA , Mohseni‐Bandpei MA , Keshtkar AA . Motor control exercise for symptomatic lumbar disc herniation: protocol for a systematic review and meta‐analysis. BMJ Open. 2016;6:e012426. doi:10.1136/bmjopen-2016-012426 PMC5051468 27678542

[34] Singh V , Malik M , Kaur J , Kulandaivelan S , Punia S . A systematic review and meta‐analysis on the efficacy of physiotherapy intervention in management of lumbar prolapsed intervertebral disc. Int J Health Sci (Qassim). 2021;15:49‐57. 33708044 PMC7934127

[35] Swanson BT . Tandem spinal stenosis: a case of stenotic cauda equina syndrome following cervical decompression and fusion for spondylotic cervical myelopathy. J Man Manip Ther. 2012;20:50‐56. doi:10.1179/2042618611Y.0000000010 23372394 PMC3267447

[36] Podnar S . Cauda equina lesions as a complication of spinal surgery. Eur Spine J. 2010;19:451‐457. 19768646 10.1007/s00586-009-1170-y PMC2899755

[37] Hoeritzauer I , Pronin S , Carson A , Statham P , Demetriades AK , Stone J . The clinical features and outcome of scan‐negative and scan‐positive cases in suspected cauda equina syndrome: a retrospective study of 276 patients. J Neurol. 2018;265:2916‐2926. doi:10.1007/s00415-018-9078-2 30298195 PMC6244667

[38] Hoeritzauer I , Carson A , Statham P , et al. Scan‐negative cauda equina syndrome: a prospective cohort study. Neurology. 2021;96:e433‐e447. 33177221 10.1212/WNL.0000000000011154

[39] Puentedura EJ , Slaughter R , Reilly S , Ventura E , Young D . Thrust joint manipulation utilization by U.S. physical therapists. J Man Manip Ther. 2017;25:74‐82. doi:10.1080/10669817.2016.1187902 28559666 PMC5430452

[40] Greenhalgh S , Finucane L , Mercer C , Selfe J . Assessment and management of cauda equina syndrome. Musculoskelet Sci Pract. 2018;37:69‐74. doi:10.1016/j.msksp.2018.06.002 29935940

[41] Billington J , Baker A . Is there a relationship between prescribed medications and symptoms of cauda equina syndrome in patients with evidence of degenerative change in the lumbar spine? Spine J. 2015;15:S57. doi:10.1016/j.spinee.2014.12.050

[42] Crisafulli S , Cutroneo PM , Verhamme K , et al. Drug‐induced urinary retention: an analysis of a national spontaneous adverse drug reaction reporting database. Eur Urol Focus. 2022;8:1424‐1432. doi:10.1016/j.euf.2021.07.001 34275763

[43] Fuso FAF , Dias ALN , Letaif OB , Cristante AF , Marcon RM , de Barros Filho TEP . Epidemiological study of cauda equina syndrome. Acta Ortop Bras. 2013;21:159‐162. 24453661 10.1590/S1413-78522013000300006 PMC3861993

[44] Planty‐Bonjour A , Kerdiles G , François P , et al. Cauda equina syndrome: poor recovery prognosis despite early treatment. Spine. 2022;47:105. doi:10.1097/BRS.0000000000004170 34265807

[45] König A , Amelung L , Danne M , Meier U , Lemcke J . Do we know the outcome predictors for cauda equine syndrome (CES)? A retrospective, single‐center analysis of 60 patients with CES with a suggestion for a new score to measure severity of symptoms. Eur Spine J. 2017;26:2565‐2572. doi:10.1007/s00586-017-5131-6 28526917

[46] Comer C , Finucane L , Mercer C , Greenhalgh S . SHADES of grey – the challenge of ‘grumbling’ cauda equina symptoms in older adults with lumbar spinal stenosis. Musculoskelet Sci Pract. 2020;45:102049. doi:10.1016/j.msksp.2019.102049 31439453

[47] Woodfield J , Lammy S , Jamjoom AAB , et al. Demographics of cauda equina syndrome: a population‐based incidence study. Neuroepidemiology. 2022;56:460‐468. doi:10.1159/000527727 36315989 PMC9945186

[48] R Core Team . R: A Language and Environment for Statistical Computing. R Foundation for Statistical Computing, Vienna, Austria; 2022.

[49] Signorell A . DescTools: Tools for Descriptive Statistics. 2024.

[50] Wickham H . ggplot2: Elegant Graphics for Data Analysis. Springer‐Verlag New York; 2016.

[51] Shi X , Miao W , Tchetgen ET . A selective review of negative control methods in epidemiology. Curr Epidemiol Rep. 2020;7:190‐202. doi:10.1007/s40471-020-00243-4 33996381 PMC8118596

[52] Levintow SN , Nielson CM , Hernandez RK , et al. Pragmatic considerations for negative control outcome studies to guide non‐randomized comparative analyses: a narrative review. Pharmacoepidemiol Drug Saf. 2023;32:599‐606. doi:10.1002/pds.5623 36965103

[53] Nordahl‐Hansen A , Øien RA , Volkmar F , Shic F , Cicchetti DV . Enhancing the understanding of clinically meaningful results: a clinical research perspective. Psychiatry Res. 2018;270:801‐806. doi:10.1016/j.psychres.2018.10.069 30551328

[54] Gatterman MI . Standards of practice relative to complications of and contraindications to spinal manipulative therapy. J Can Chiropr Assoc. 1991;35:232‐236.

[55] Chu E . WHO Guidelines on Basic Training and Safety on Chiropractic. World Health Organization; 2005.

[56] Chu E , Trager R . Prevalence of serious pathology among adults with low Back pain presenting for chiropractic care: a retrospective chart review of integrated clinics in Hong Kong. Med Sci Monit. 2022;28:e938042. doi:10.12659/MSM.938042 36164262 PMC9526366

[57] Daniel DM , Ndetan H , Rupert RL , Martinez D . Self‐reported recognition of undiagnosed life threatening conditions in chiropractic practice: a random survey. Chiropr Man Therap. 2012;20:1‐6. doi:10.1186/2045-709X-20-21 PMC3431984 22764778

[58] Forrest JB , Moldwin R . Diagnostic options for early identification and management of interstitial cystitis/painful bladder syndrome. Int J Clin Pract. 2008;62:1926‐1934. doi:10.1111/j.1742-1241.2008.01931.x 19166439

[59] Bergstra SA , Sepriano A , Ramiro S , Landewé R . Three handy tips and a practical guide to improve your propensity score models. RMD Open. 2019;5:e000953. doi:10.1136/rmdopen-2019-000953 31168417 PMC6525599

[60] Ehrler M , Peterson C , Leemann S , Schmid C , Anklin B , Humphreys BK . Symptomatic, MRI confirmed, lumbar disc herniations: a comparison of outcomes depending on the type and anatomical axial location of the hernia in patients treated with high‐velocity, low‐amplitude spinal manipulation. J Manip Physiol Ther. 2016;39:192‐199. doi:10.1016/j.jmpt.2016.02.013 27034106

[61] Leemann S , Peterson CK , Schmid C , Anklin B , Humphreys BK . Outcomes of acute and chronic patients with magnetic resonance imaging–confirmed symptomatic lumbar disc herniations receiving high‐velocity, low‐amplitude, spinal manipulative therapy: a prospective observational cohort study with one‐year follow‐up. J Manip Physiol Ther. 2014;37:155‐163. doi:10.1016/j.jmpt.2013.12.011 24636109

[62] Chu EC‐P , Trager RJ , Lee LY‐K , Niazi IK . A retrospective analysis of the incidence of severe adverse events among recipients of chiropractic spinal manipulative therapy. Sci Rep. 2023;13:1254. doi:10.1038/s41598-023-28520-4 36690712 PMC9870863

[63] Trager RJ , Daniels CJ , Perez JA , Casselberry RM , Dusek JA . Association between chiropractic spinal manipulation and lumbar discectomy in adults with lumbar disc herniation and radiculopathy: retrospective cohort study using United States' data. BMJ Open. 2022;12:e068262. doi:10.1136/bmjopen-2022-068262 PMC9764600 36526306

[64] Zhang W , Guo W , Zhao P , et al. Therapeutic effects of Chinese osteopathy in patients with lumbar disc herniation. Am J Chin Med. 2013;41:983‐994. 24117063 10.1142/S0192415X13500663

[65] Harwood KJ , Pines JM , Andrilla CHA , Frogner BK . Where to start? A two stage residual inclusion approach to estimating influence of the initial provider on health care utilization and costs for low back pain in the US. BMC Health Serv Res. 2022;22:694. doi:10.1186/s12913-022-08092-1 35606781 PMC9128255

[66] Clijsters M , Fronzoni F , Jenkins H . Chiropractic treatment approaches for spinal musculoskeletal conditions: a cross‐sectional survey. Chiropr Man Therap. 2014;22:1‐10. doi:10.1186/s12998-014-0033-8 PMC4193988 25309722

[67] Kreiner DS , Hwang SW , Easa JE , et al. An evidence‐based clinical guideline for the diagnosis and treatment of lumbar disc herniation with radiculopathy. Spine J. 2014;14:180‐191. doi:10.1016/j.spinee.2013.08.003 24239490

[68] Stochkendahl MJ , Kjaer P , Hartvigsen J , et al. National clinical guidelines for non‐surgical treatment of patients with recent onset low back pain or lumbar radiculopathy. Eur Spine J. 2018;27(1):60‐75. 28429142 10.1007/s00586-017-5099-2

[69] Bussieres AE , Stewart G , Al‐Zoubi F , et al. Spinal manipulative therapy and other conservative treatments for low back pain: a guideline from the Canadian chiropractic guideline initiative. J Manip Physiol Ther. 2018;41:265‐293. doi:10.1016/j.jmpt.2017.12.004 29606335

[70] Chou R , Côté P , Randhawa K , et al. The Global Spine Care Initiative: applying evidence‐based guidelines on the non‐invasive management of back and neck pain to low‐and middle‐income communities. Eur Spine J. 2018;27:851‐860. 10.1007/s00586-017-5433-8 29460009

[71] National Guideline Centre (UK) . Low Back Pain and Sciatica in Over 16s: Assessment and Management. National Institute for Health and Care Excellence (NICE); 2020. 33090750

[72] National Guideline Centre (UK) : Low Back Pain and Sciatica in Over 16s: Assessment and Management. National Institute for Health and Care Excellence (NICE); 2016. 27929617

[73] Hegmann KT , Travis R , Andersson GBJ , et al. Non‐invasive and minimally invasive management of low back disorders. J Occup Environ Med. 2020;62:e111. doi:10.1097/JOM.0000000000001812 31977923

[74] Trager RJ , Bejarano G , Perfecto R‐PT , Blackwood ER , Goertz CM . Chiropractic and spinal manipulation: a review of research trends, evidence gaps, and guideline recommendations. J Clin Med. 2024;13:5668. doi:10.3390/jcm13195668 39407729 PMC11476883

[75] Schneider MJ , Ammendolia C , Murphy DR , et al. Comparative clinical effectiveness of nonsurgical treatment methods in patients with lumbar spinal stenosis: a randomized clinical trial [with consumer summary]. JAMA Netw Open. 2019;2(1):e186828. 30646197 10.1001/jamanetworkopen.2018.6828 PMC6324321

[76] Linked EHR and Claims. TriNetX: Revealing the Patient Journey with Linked Claims and EHR. Accessed May 17, 2025 https://trinetx.com/real-world-data/linked/

[77] Malek S , Stein E , Swartzbaugh M , Brown J . RWD68 the TriNetX linked EHR and administrative claims network. Value Health. 2024;27:S370. doi:10.1016/j.jval.2024.03.1722

