# Supplementary material for: Chiropractic spinal manipulative therapy versus physical therapist‐led exercise and the risk of cauda equina syndrome in adults with lumbar disc herniation, stenosis, or radiculopathy
Source: PM R. 2026 Jan 3;18(7):723–34. doi: 10.1002/pmrj.70071 (PMC13105361; doi:10.1002/pmrj.70071)
Supplement: Supplementary file 1 — Data S1. Supporting Information. [file PMRJ-18-723-s001.pdf]

Supplemental File

For: "Chiropractic spinal manipulative therapy versus physical therapist-led exercise and the risk of cauda equina syndrome in adults with lumbar disc herniation, stenosis, or radiculopathy"

Robert J. Trager, Anthony N. Baumann, Romeo-Paolo T. Perfecto, Christine M. Goertz

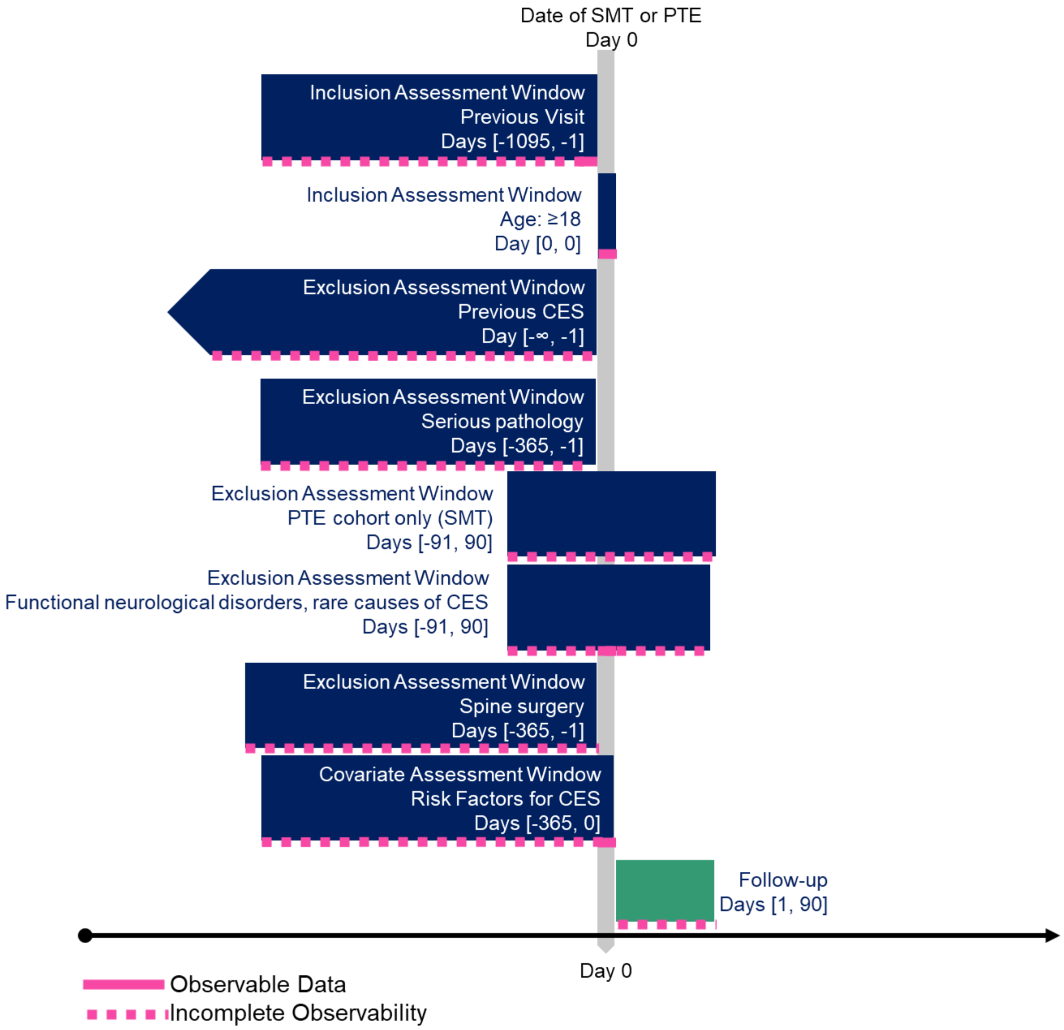

Figure S1: Visualization of study design. The vertical grey arrow represents the index date whereby patients receive spinal manipulative therapy (SMT) or physical therapist-led therapeutic exercise (PT). The boxes and related text indicate a specified number of days ([#, #]) relative to the index date whereby eligibility criteria were applied. The exclusion for previous cauda equina syndrome (CES) spanned as long as preceding data were available per patient (-∞). Figure created by Robert Trager using a Creative Commons template [1].

Table S1: Inclusion codes for both cohorts

**Diagnosis codes***	**Definition**
G54.4	Lumbosacral root disorders, not elsewhere classified
M47.26	Other spondylosis with radiculopathy, lumbar region
M47.27	Other spondylosis with radiculopathy, lumbosacral region
M48.06	Spinal stenosis, lumbar region
M48.07	Spinal stenosis, lumbosacral region
M51.16	Intervertebral disc disorders with radiculopathy, lumbar region
M51.17	Intervertebral disc disorders with radiculopathy, lumbosacral region
M51.26	Other intervertebral disc displacement, lumbar region (i.e., disc herniation)
M51.27	Other intervertebral disc displacement, lumbosacral region (i.e., disc herniation)
M54.16	Radiculopathy, lumbar region
M54.17	Radiculopathy, lumbosacral region
M54.18	Radiculopathy, sacral and sacrococcygeal region
M54.3	Sciatica
M54.4	Lumbago with sciatica
*International Classification of Diseases, 10th Revision (ICD-10)	

Table S2: Exclusion criteria for both cohorts

Code	Definition	Days
G54.1, G81, G82.2, G83.4, G95.81, M51.0, S24, S34 (ICD-10)	CES & related conditions: Lumbosacral plexus disorders; paraplegia; cauda equina syndrome; conus medullaris syndrome; thoracic, thoracolumbar and lumbosacral intervertebral disc disorders with myelopathy; other and unspecified injury of lumbar and sacral spinal cord; injury of nerves and spinal cord at thorax level; injury of lumbar and sacral spinal cord and nerves at abdomen, lower back and pelvis level	-∞ to -1
R53.2, F44.4, F44.5, F44.6, F44.7, Z76.5 (ICD-10)	Functional neurological disorders and conscious simulation: Functional quadriplegia; conversion disorder with motor or sensory deficit, seizures, or mixed presentations; malingerer	-91 to 91
A52.11, B00.3, B02.2, B27.12, G61.0, I74.09 (ICD-10)	Rare etiologies or mimickers of CES: Tabes dorsalis, herpesviral meningitis; Zoster with nervous system involvement; cytomegaloviral mononucleosis with meningitis; other arterial embolism and thrombosis of abdominal aorta; Guillain-Barré syndrome	-91 to 91
A17, A18.01, G00-G09, M46.2, M46.3, M46.4, M46.5 (ICD-10)	Spinal infection: Tuberculosis of nervous system or spine; inflammatory diseases of the central nervous system (e.g., abscess); osteomyelitis of vertebra, infection of intervertebral disc (pyogenic), discitis, or other infective spondylopathies	-365 to -1
C41.2, C41.4, C70.1, C72.0, C72.1, C79.4, C79.5, C80.0, D33.4, D32.1, D42.1, D43.4 (ICD-10)	Spinal neoplasm: Malignant neoplasm of vertebral column, pelvic bones, sacrum, and coccyx, spinal cord, spinal meninges, unspecified part of nervous system, or cauda equina; secondary malignant neoplasm of bone and bone marrow; benign neoplasm of spinal cord; benign neoplasm of spinal meninges; neoplasm of uncertain behavior of spinal meninges; neoplasm of uncertain behavior of spinal cord	-365 to -1
M48.46, M48.56, M48.57, M80.08, M80.88, M80.8B, S22.0, S28, S38, S32 (ICD-10)	Spinal fracture: Fatigue fracture of vertebra, lumbar region; collapsed vertebra, lumbar region or lumbosacral region; age-related osteoporosis with current pathological fracture, vertebra(e) other osteoporosis with current pathological fracture, vertebra(e) or pelvis; fracture of thoracic vertebra; crushing injury of thorax, and traumatic amputation of part of thorax; crushing injury and traumatic amputation of abdomen, lower back, pelvis and external genitals; fracture of lumbar spine and pelvis	-365 to -1
Q05.9, Q06.3 (ICD-10)	Congenital disorders: Spina bifida, other congenital malformations of cauda equina	-∞to -1
S06.4, S06.5, S06.6 (ICD-10)	Spinal vascular pathology: Epidural hemorrhage, traumatic subdural hemorrhage, or traumatic subarachnoid hemorrhage	-365 to -1
N31, N39.490, N39.498, N39.490, R32, R33, R34, R35, T79.5	Bladder dysfunction: Neuromuscular dysfunction of the bladder; other specified urinary incontinence (i.e., reflex/total); overflow incontinence; unspecified urinary incontinence; retention of urine; anuria and oliguria; polyuria; traumatic anuria	-365 to -1
R15, K59.2 (ICD-10)	Fecal incontinence: Fecal incontinence; neurogenic bowel, not elsewhere classified	-365 to -1
0T9B, 0T9C, 0T9D (ICD-10-PCS); 429723008, 720649006 (SNOMED), 51701, 1008272 [51702, 51703] (CPT), T83.0 (ICD-10)	Urinary catheterization: Bladder, bladder neck, and urethra (drainage); procedure involving urinary catheter; introduction of urinary catheter; insertion of non-indwelling bladder catheter; insertion of temporary indwelling bladder catheter, mechanical complication of urinary catheter	-365 to -1
1004038, 1009340, 1002886 (CPT), 3E0R, 3E0S (ICD-10-PCS)	Spine surgery and injection: Surgical procedures on the spine or spinal cord (including injections); anesthesia for procedures on the spine and spinal cord; physiological systems introduction, spinal canal and epidural space	-91 to -1
97140, 1013558, 98940, 98941, 98942 (CPT)	Additional exclusions for the physical therapy cohort: Manual therapy techniques (e.g., mobilization/manipulation, manual traction); osteopathic manipulative treatment procedures; chiropractic manipulative treatment, spinal	-91 to 91
Abbreviations: Current Procedural Terminology (CPT); Healthcare Common Procedure Coding System (HCPCS); International Classification of Diseases, 10th Revision (ICD-10) and ICD-10 Procedure Coding System (ICD-10-PCS), as far retrospective in the patient's chart as data are available (-∞), Systematized Nomenclature of Medicine (SNOMED)		

Table S3: Variables controlled for using propensity score matching

Variable/Code	Description	References
Demographics	Age, sex	[2,3]
Comorbidities associated with CES		
E08-E13	Diabetes mellitus	[2,4]
E66	Overweight and obesity	[5,6]
F30-F39	Mood disorders	[3,7,8]
F40-F48	Anxiety, dissociative, stress-related, somatoform and other nonpsychotic mental disorders	[3,7,8]
G20	Parkinson's disease	[7,9]
G35	Multiple sclerosis	[3,4,7,10]
G35-G37	Demyelinating diseases of the central nervous system	[3,4,7,10]
G60-G65	Polyneuropathies and other disorders of the peripheral nervous system	[11,12]
G61	Inflammatory polyneuropathy	[11,12]
G83	Other paralytic syndromes	[13]
G61.81	Chronic inflammatory demyelinating polyneuritis	[11,12]
G89.2	Chronic pain, not elsewhere classified	[3,7,8]
G95	Other and unspecified diseases of spinal cord	[10]
I60-I69	Cerebrovascular diseases	[7,9,9,14,15]
M21.37	Foot drop, acquired	[16]
M43.16	Spondylolisthesis, lumbar region	[13]
M45	Ankylosing spondylitis	[17–19]
M46	Other inflammatory spondylopathies	[17–19]
M48.06	Spinal stenosis, lumbar region	[6,17,20–22]
M48.07	Spinal stenosis, lumbosacral region	[6,17,20–22]
M79.7	Fibromyalgia	[3]
M96.1	Postlaminectomy syndrome, not elsewhere classified	[23,24]
Urinary disorders or symptoms		
N30-N39	Other diseases of the urinary system	[25,26]
N40	Benign prostatic hyperplasia	[4,27]
R30-R39	Symptoms and signs involving the genitourinary system (e.g., difficulties with micturition)	[25,26]
Trauma		
S20-S29	Injuries to the thorax	[10]
S30-S39	Injuries to the abdomen, lower back, lumbar spine, pelvis and external genitals	[25]
T14	Injury of unspecified body region	[2,26,28]
V00-Y99	External causes of morbidity (e.g., motor vehicle collision)	[2,26,28]
Medications associated with neuropathic bladder or urinary retention, also used for low back pain		
CN101 (VA)	Opioid analgesics	[4,7,8,29,30]
CN302 (VA)	Benzodiazepine derivative sedatives/hypnotics	[4,7,8,29,30]
MS200 (VA)	Skeletal muscle relaxants	[15]
N02BF (ATC)	Gabapentinoids	[4,7,8,29,30]
CN601 (VA)	Tricyclic antidepressants	[4,7,8,29,30]

Abbreviations: Anatomical Therapeutic Chemical (ATC); Current Procedural Terminology (CPT); cauda equina syndrome (CES); International Classification of Diseases, 10th Edition (ICD-10); Logical Observation Identifiers Names and Codes (LOINC); Veterans Health Administration National Drug File (VA), custom TriNetX code (*)

Table S4: Physical therapy follow-up visit codes

Code	Definition
1013490 (CPT)	Physical medicine and rehabilitation modalities (e.g., therapeutic ultrasound, electrical stimulation, mechanical traction)
1013510 (CPT)	Physical medicine and rehabilitation therapeutic procedures (e.g., therapeutic exercise)
91251008 (SNOMED)	Physical therapy procedure
F07 (ICD-10-PCS)	Motor treatment (e.g., therapeutic exercise)
G0283 (HCPCS)	Electrical stimulation (unattended), to one or more areas for indication(s) other than wound care, as part of a therapy plan of care
398074008 (SNOMED)	Thermotherapy with hot packs
20560 (CPT)	Needle insertion(s) without injection(s); 1 or 2 muscle(s)
20561 (CPT)	Needle insertion(s) without injection(s); 3 or more muscles
97799 (CPT)	Unlisted physical medicine/rehabilitation service or procedure
97002 (CPT)	Physical therapy re-evaluation (deprecated 2018)
97164 (CPT)	Re-evaluation of physical therapy established plan of care, requiring these components: An examination including a review of history and use of standardized tests and measures is required; and Revised plan of care using a standardized patient assessment instrument and/or measurable assessment of functional outcome Typically, 20 minutes are spent face-to-face with the patient and/or family
Abbreviations: Current Procedural Terminology (CPT); International Classification of Diseases, 10th Revision Procedure Coding System (ICD-10-PCS) Systematized Nomenclature of Medicine (SNOMED)	

Table S5: Negative control outcomes

Variable	Definition
1022231 (CPT)	Colonoscopy
18631 (CPT)	Azithromycin
J00-J06 (ICD-10)	Acute upper respiratory infections
K35 (ICD-10)	Acute appendicitis

Abbreviations: Current Procedural Terminology (CPT); International Classification of Diseases, 10th Revision (ICD-10); vaccine administered code set (CVX).

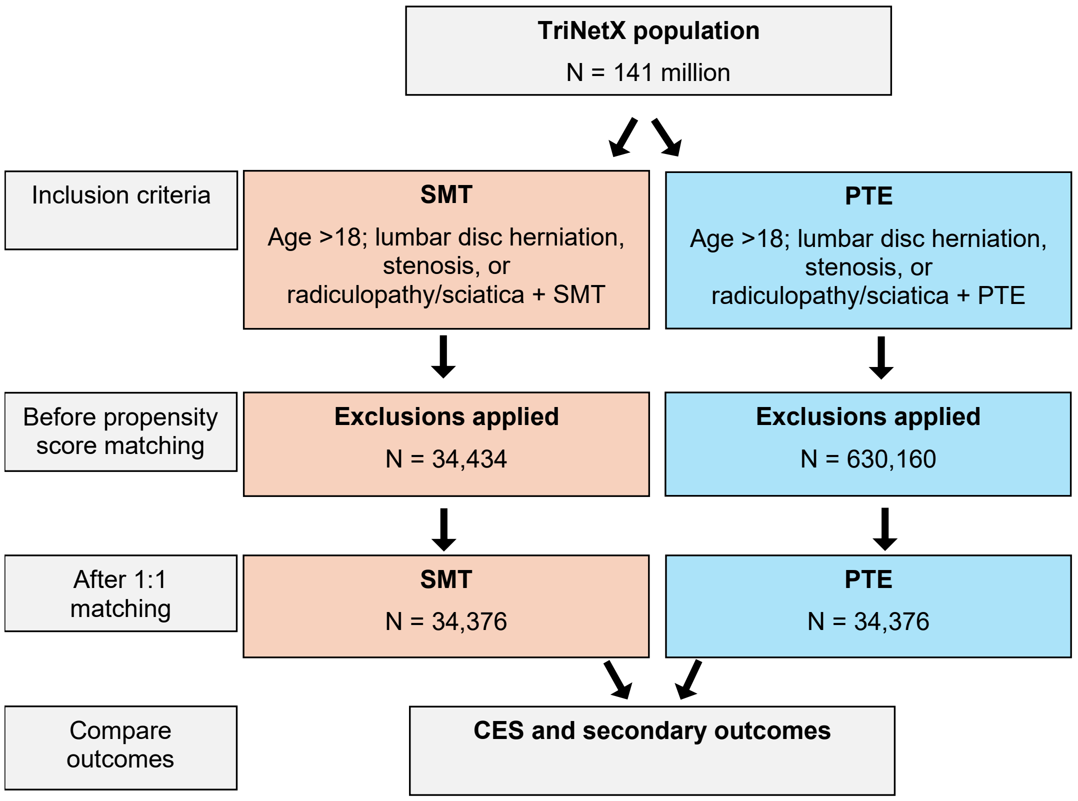

Figure S2: Patient selection flowchart. In the physical therapist-led exercise (PTE) cohort, exclusions from the base population (defined by inclusionary criteria) were 22% for recent spinal manipulative therapy (SMT), 12% for lack of follow-up visit, 3% for no preceding visit, 1% for pre-existing cauda equina syndrome (CES) and related conditions, 1% for pre-existing serious pathology, and 1% for recent spine surgery. In the SMT cohort, exclusions were 7% for no preceding visit, 5% for lack of follow-up visit, 2% for pre-existing CES and related conditions, and 1% for recent spine surgery. These values represent trimming prior to matching, after which additional trimming occurred as unmatched patients were discarded (predominantly affecting the larger PTE cohort).

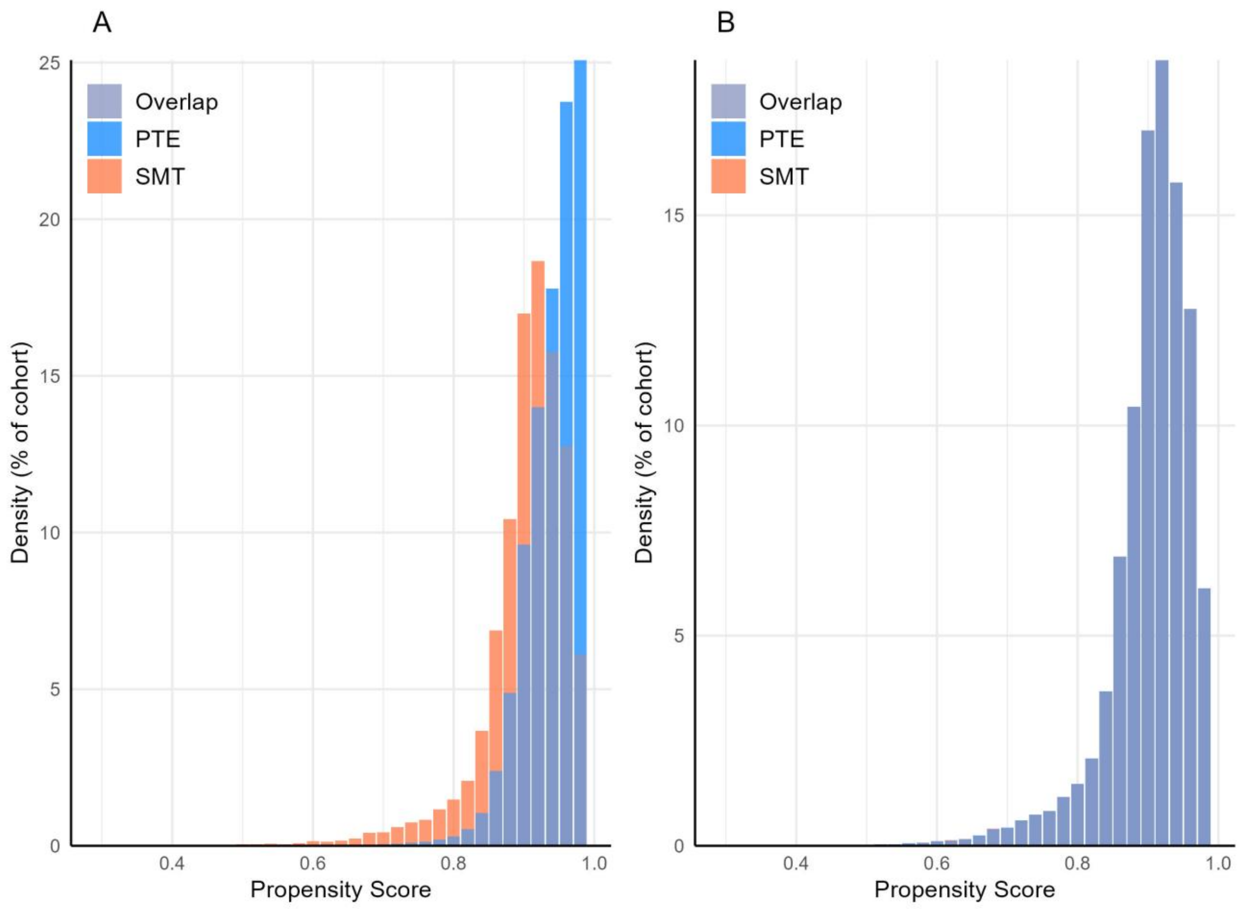

Figure S3: Propensity score density graph. Density scores are shown before (A) and after (B) matching. The orange bars indicate the spinal manipulative therapy (SMT) cohort while the light blue bars represent the physical therapist-led therapeutic exercise (PTE) cohort. Densities are superimposed after matching, highlighted by a darker blue color, suggesting sufficient balance of covariates.

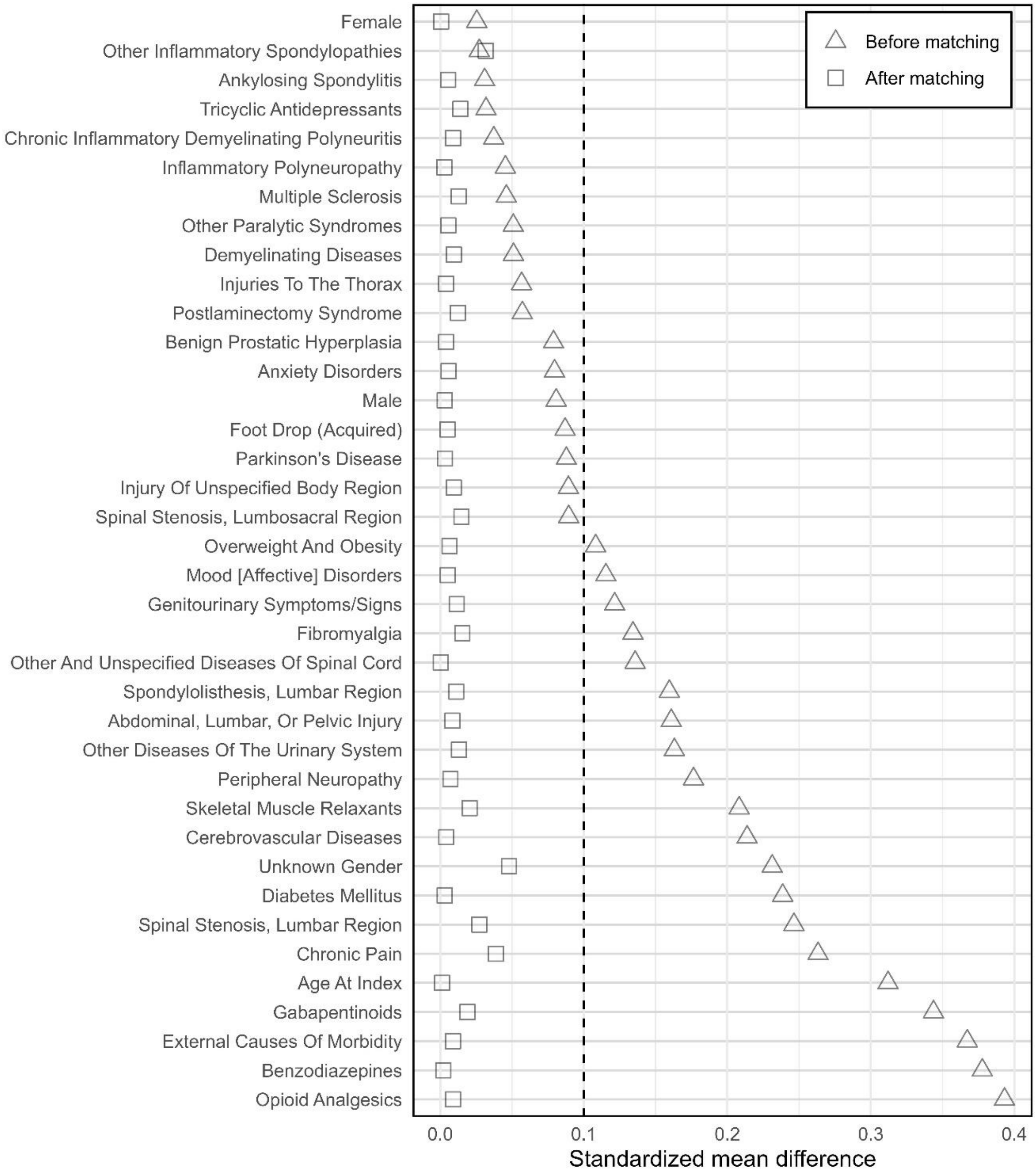

Figure S4: Covariate balance plot. Standardized mean differences (SMDs) for baseline characteristics are shown both before (triangles) and after matching (squares). The vertical dashed line at SMD=0.1 represents the a priori threshold for covariate balance, with points exceeding this being meaningfully imbalanced [31,32]. All covariates have adequate balance after matching. Plot created by Robert Trager using *R and R studio (version 4.2.2, Vienna, AT [33]) and the ggplot2 package [34].*

Table S6: Detailed follow-up metrics. Values are shown for the spinal manipulative therapy (SMT) and physical therapist-led therapeutic exercise (PTE) cohorts after propensity score matching.

Metric	SMT	PTE
Total patients	34376	34376
Mean follow-up (SD) days	88.6 (8.7)	87.5 (11.8)
Median follow-up days (interquartile range)	90 (0)	90 (0)
Years of observation (entire cohort)	8341	8240
Patients with complete follow-up (%)	96.7	94.5

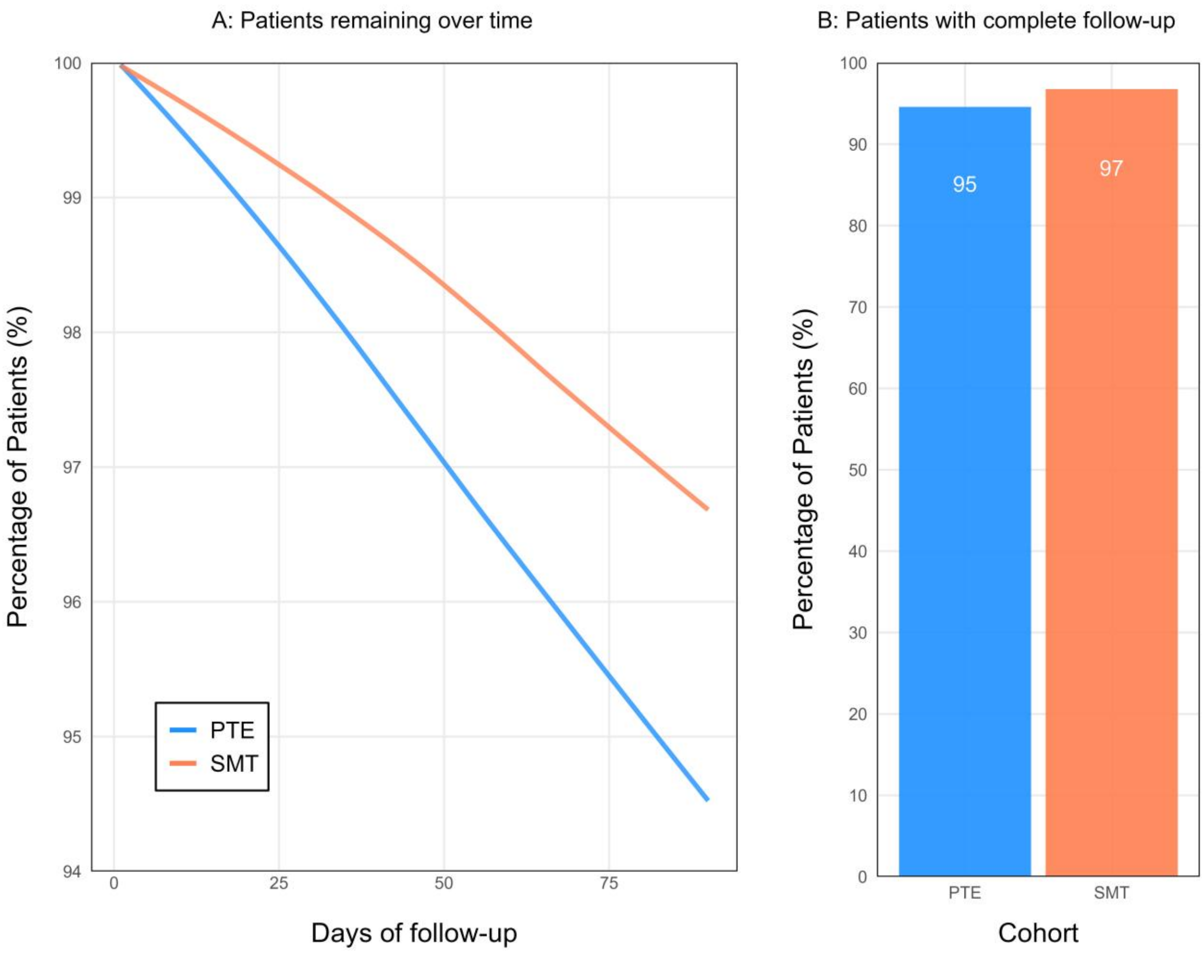

Figure S5: Proportion of patients remaining as a function of time. Figure A shows the percentage of patients who have follow up at least as far as that date in each cohort. This plot uses locally estimated scatterplot smoothing. Figure B bars show the percentage of patients who remained for at least the maximum follow-up time of 90 days. In both plots, the spinal manipulative therapy (SMT) cohort is shown in orange, while the physical therapist-led therapeutic exercise (PTE) cohort is shown in blue. Both plots were created by Robert J. Trager using R and R studio (version 4.2.2, Vienna, AT [24]) and the ggplot2 package [25].

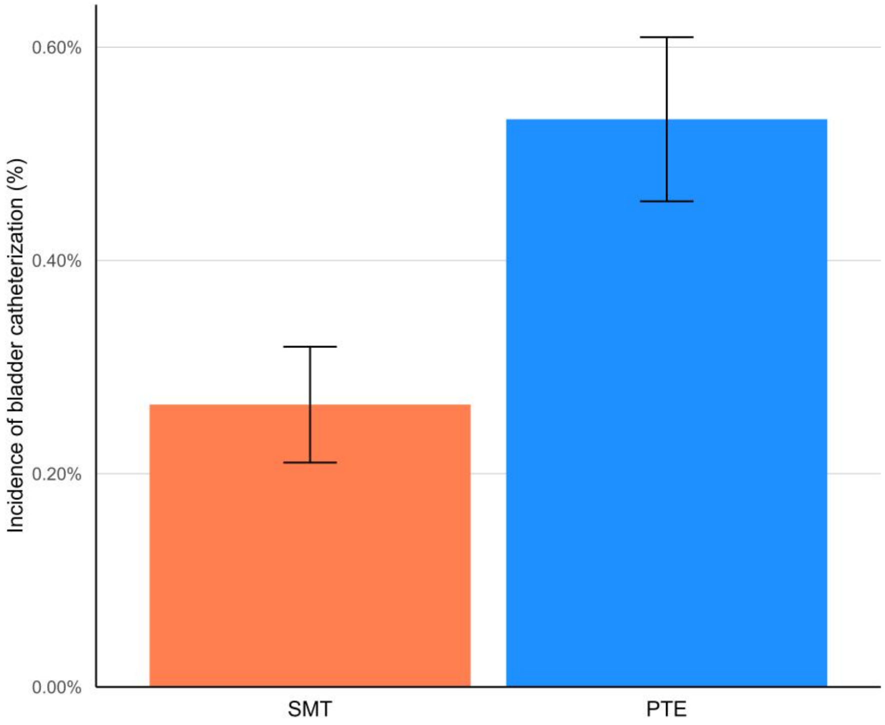

Figure S6: Total incidences of bladder catheterization per cohort over a 90-day follow-up window. The incidence of CES in the cohort receiving spinal manipulative therapy (SMT) is shown in orange, while the incidence of CES in the cohort receiving physical therapist-led therapeutic exercise (PTE) is shown in blue. The error bars demonstrate 95% confidence intervals.

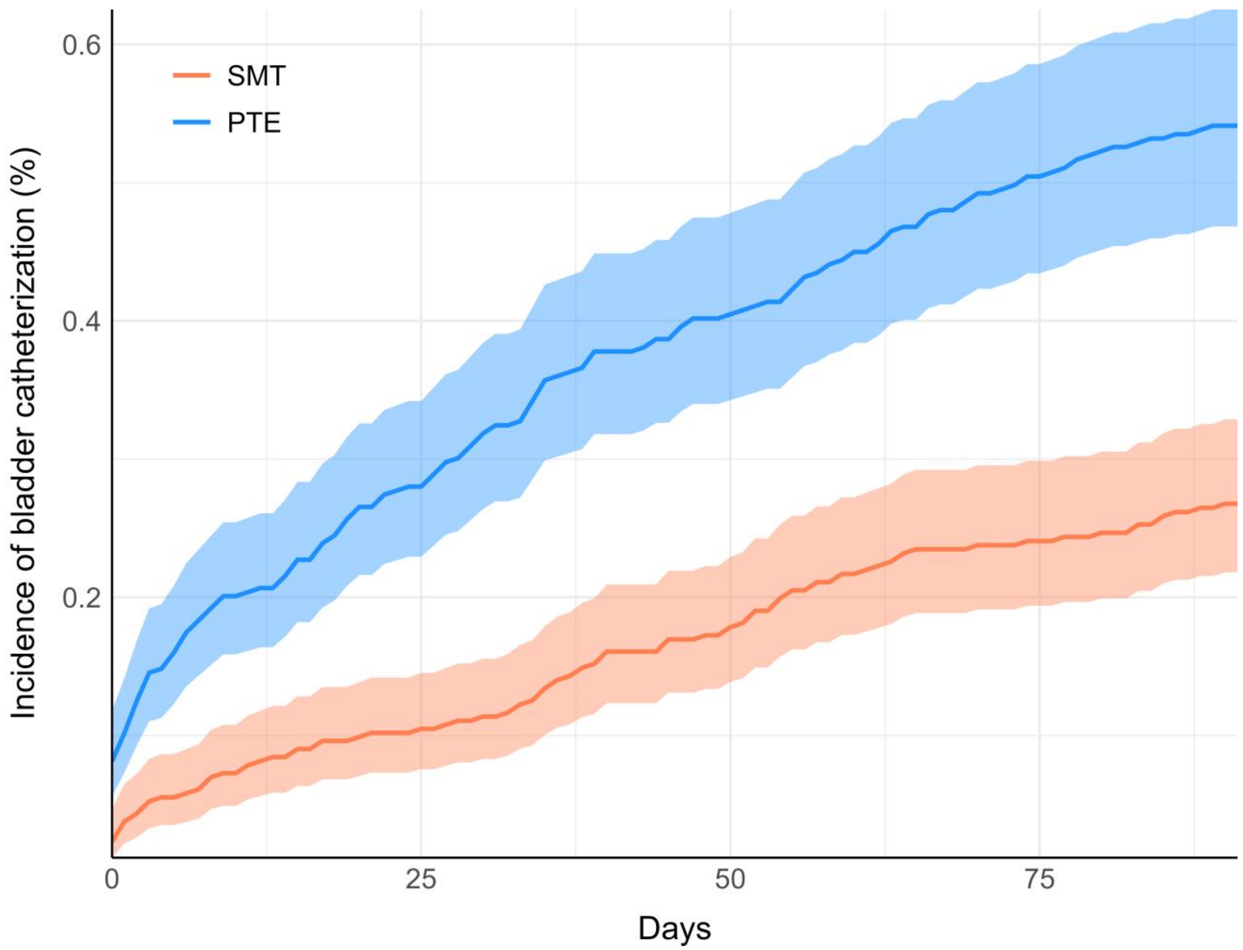

Figure S7: Cumulative incidence of bladder catheterization per cohort over a 90-day follow-up window. The incidence of CES in the cohort receiving spinal manipulative therapy (SMT) is shown in orange, while the incidence of CES in the cohort receiving physical therapist-led therapeutic exercise (PTE) is shown in blue. The shaded regions demonstrate 95% confidence intervals.

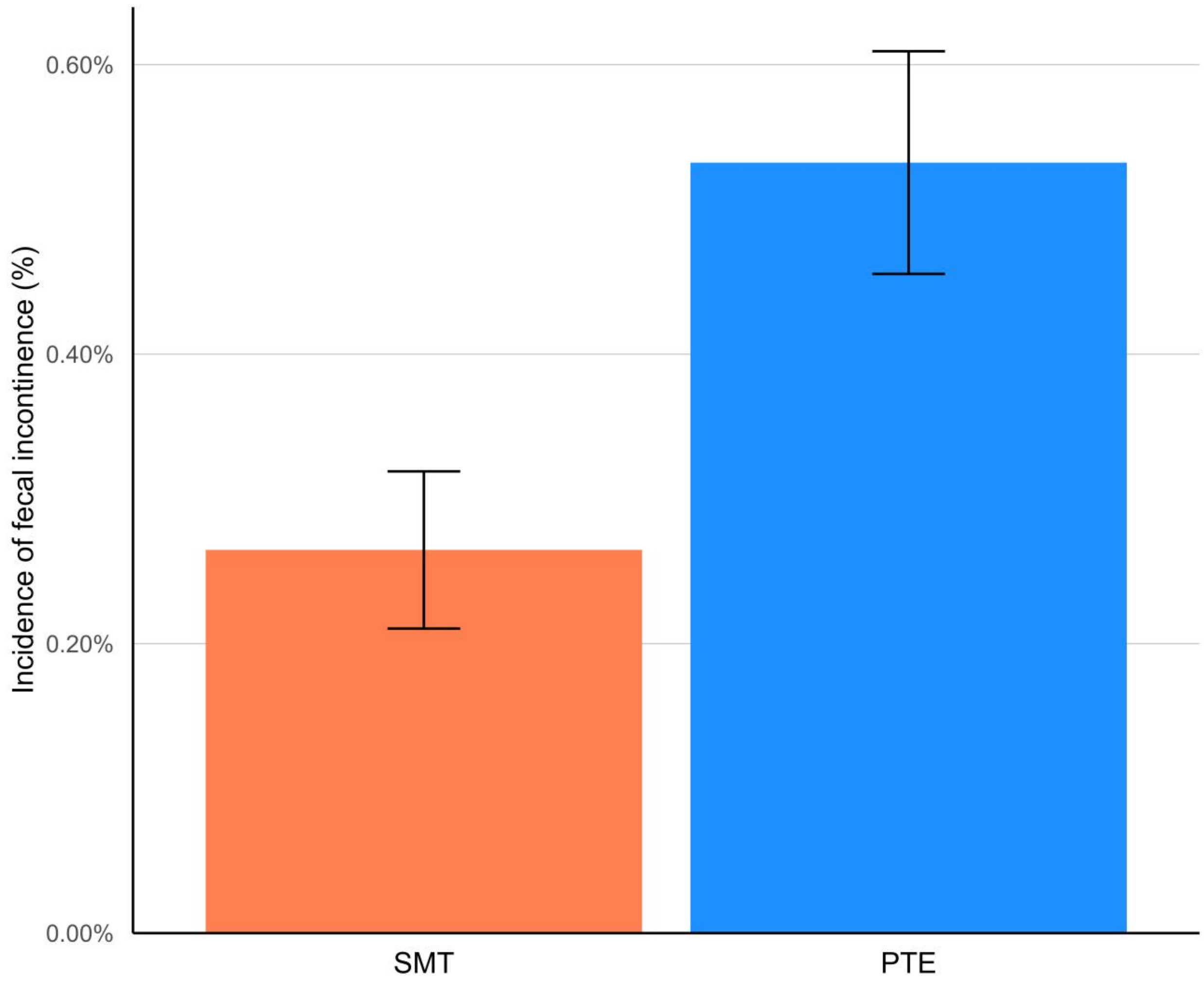

Figure S8: Total incidences of fecal incontinence per cohort over a 90-day follow-up window. The incidence of CES in the cohort receiving spinal manipulative therapy (SMT) is shown in orange, while the incidence of CES in the cohort receiving physical therapist-led therapeutic exercise (PTE) is shown in blue. The error bars demonstrate 95% confidence intervals.

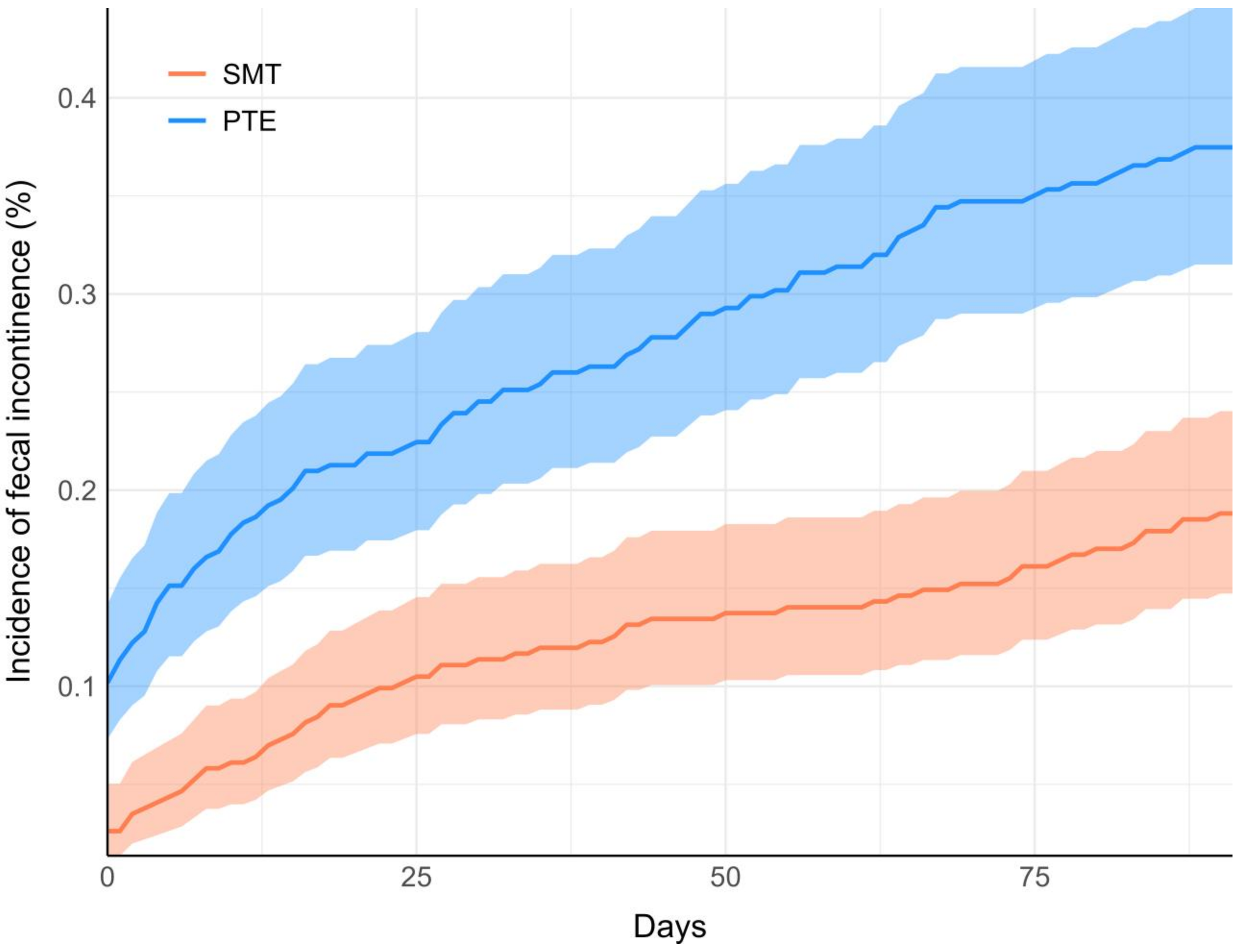

Figure S9: Cumulative incidence of fecal incontinence per cohort over a 90-day follow-up window. The incidence of CES in the cohort receiving spinal manipulative therapy (SMT) is shown in orange, while the incidence of CES in the cohort receiving physical therapist-led therapeutic exercise (PTE) is shown in blue. The shaded regions demonstrate 95% confidence intervals.

References

1. Wang SV, Schneeweiss S. A Framework for Visualizing Study Designs and Data Observability in Electronic Health Record Data. Clin Epidemiol. 2022;14:601–8.

2. Marascalchi BJ, Passias PG, Goz V, Weinreb JH, Joo L, Errico TJ. Comparative Analysis of Patients With Cauda Equina Syndrome Versus an Unaffected Population Undergoing Spinal Surgery. Spine. 2014;39:482.

3. Gibson LL, Harborow L, Nicholson T, Bell D, David AS. Is scan-negative cauda equina syndrome a functional neurological disorder? A pilot study. Eur J Neurol. 2020;27:1336–42.

4. Greenhalgh S, Finucane L, Mercer C, Selfe J. Assessment and management of cauda equina syndrome. Musculoskelet Sci Pract. 2018;37:69–74.

5. Venkatesan M, Uzoigwe CE, Perianayagam G, Braybrooke JR, Newey ML. Is cauda equina syndrome linked with obesity? J Bone Joint Surg Br. 2012;94-B:1551–6.

6. Cushnie D, Urquhart JC, Gurr KR, Siddiqi F, Bailey CS. Obesity and spinal epidural lipomatosis in cauda equina syndrome. Spine J. 2018;18:407–13.

7. Hoeritzauer I, Pronin S, Carson A, Statham P, Demetriades AK, Stone J. The clinical features and outcome of scan-negative and scan-positive cases in suspected cauda equina syndrome: a retrospective study of 276 patients. J Neurol [Internet]. 2018 [cited 2018 Nov 3]; Available from: https://doi.org/10.1007/s00415-018-9078-2

8. Hoeritzauer I, Carson A, Statham P, Panicker JN, Granitsiotis V, Eugenicos M, et al. Scan-negative cauda equina syndrome: a prospective cohort study. Neurology. 2021;96:e433–47.

9. McDonnell J, Ahern DP, Gibbons D, Dalton DM, Butler JS. A systematic review of the presentation of scan-negative suspected cauda equina syndrome. Surg J R Coll Surg Edinb Irel. 2020;18:49–52.

10. Stolper K, Haug JC, Christensen CT, Samsey KM, April MD. Prevalence of thoracic spine lesions masquerading as cauda equina syndrome: yield of a novel magnetic resonance imaging protocol. Intern Emerg Med. 2017;12:1259–64.

11. Chung T, Prasad K, Lloyd TE. Peripheral Neuropathy – Clinical and Electrophysiological Considerations. Neuroimaging Clin N Am. 2014;24:49–65.

12. Lewis RA. Chronic inflammatory demyelinating polyneuropathy. Neurol Clin. 2007;25:71–87.

13. Hatgis J, Hall AJ. Cauda Equina Syndrome. In: Kahn SB, Xu RY, editors. Musculoskelet Sports Spine Disord Compr Guide [Internet]. Cham: Springer International Publishing; 2017 [cited 2024 Sept 1]. p. 447–9. Available from: https://doi.org/10.1007/978-3-319-50512-1_99

14. Kumaria A, Haider Z, Ali A, Pillai D, Bommireddy R, Bateman A, et al. Intracranial Mimics of Cauda Equina Syndrome: Heads or Tails? World Neurosurg. 2020;144:e643–7.

15. Serlin DC, Heidelbaugh JJ, Stoffel JT. Urinary Retention in Adults: Evaluation and Initial Management. Am Fam Physician. 2018;98:496–503.

16. Demetriades AK, Mancuso-Marcello M, Baig Mirza A, Frantzias J, Bell DA, Selway R, et al. Acute bilateral foot drop with or without cauda equina syndrome—a case series. Acta Neurochir (Wien). 2021;163:1191–8.

17. Kuris EO, McDonald CL, Palumbo MA, Daniels AH. Evaluation and Management of Cauda Equina Syndrome. Am J Med. 2021;134:1483–9.

18. Tang C, Moser FG, Reveille J, Bruckel J, Weisman MH. Cauda Equina Syndrome in Ankylosing Spondylitis: Challenges in Diagnosis, Management, and Pathogenesis. J Rheumatol. 2019;46:1582–8.

19. López-Medina C, Moltó A. Comorbid pain in axial spondyloarthritis, including fibromyalgia. Ther Adv Musculoskelet Dis. 2020;12:1759720X20966123.

20. Kaiser R, Krajcová A, Waldauf P, Srikandarajah N, Makel M, Beneš V. Are There Any Risk Factors Associated with the Presence of Cauda Equina Syndrome in Symptomatic Lumbar Disk Herniation? World Neurosurg. 2020;141:e600–5.

21. Comer C, Finucane L, Mercer C, Greenhalgh S. SHADES of grey – The challenge of 'grumbling' cauda equina symptoms in older adults with lumbar spinal stenosis. Musculoskelet Sci Pract. 2020;45:102049.

22. Inui Y, Doita M, Ouchi K, Tsukuda M, Fujita N, Kurosaka M. Clinical and Radiologic Features of Lumbar Spinal Stenosis and Disc Herniation With Neuropathic Bladder. Spine. 2004;29:869.

23. Henriques T, Olerud C, Petrén-Mallmin M, Ahl T. Cauda Equina Syndrome as a Postoperative Complication in Five Patients Operated for Lumbar Disc Herniation. Spine. 2001;26:293.

24. Ramnarayan R, Chaurasia B. The extended post spinal surgery syndrome (EPSS): A narrative review. Romanian Neurosurg. 2024;109–16.

25. Daniels EW, Gordon Z, French K, Ahn UM, Ahn NU. Review of medicolegal cases for cauda equina syndrome: what factors lead to an adverse outcome for the provider? Orthopedics. 2012;35:e414-419.

26. Zeb J, Zaib J, Khan A, Farid M, Ambreen S, Shah SH. Characteristics and clinical features of cauda equina syndrome: insights from a study on 256 patients. SICOT-J. 9:22.

27. Imai A, Yamamoto H, Hatakeyama S, Iwabuchi I, Yoneyama T, Hashimoto Y, et al. Cauda equina symptoms are closely related to male lower urinary tract symptoms. Urol Int. 2010;84:325–9.

28. Mustafa MA, Richardson GE, Gillespie CS, Islim AI, Wilby M, Clark S, et al. Definition and surgical timing in cauda equina syndrome–An updated systematic review. PLOS ONE. 2023;18:e0285006.

29. Billington J, Baker A. Is there a relationship between prescribed medications and symptoms of cauda equina syndrome in patients with evidence of degenerative change in the lumbar spine? Spine J. 2015;15:S57.

30. Crisafulli S, Cutroneo PM, Verhamme K, Ferrajolo C, Ficarra V, Sottosanti L, et al. Drug-induced Urinary Retention: An Analysis of a National Spontaneous Adverse Drug Reaction Reporting Database. Eur Urol Focus. 2022;8:1424–32.

31. Austin PC. Balance diagnostics for comparing the distribution of baseline covariates between treatment groups in propensity-score matched samples. Stat Med. 2009;28:3083–107.

32. Stuart EA, Lee BK, Leacy FP. Prognostic score–based balance measures can be a useful diagnostic for propensity score methods in comparative effectiveness research. J Clin Epidemiol. 2013;66:S84-S90.e1.

33. R Core Team. R: A Language and Environment for Statistical Computing [Internet]. Vienna, Austria: R Foundation for Statistical Computing; 2022. Available from: https://www.R-project.org/

34. Wickham H. ggplot2: Elegant Graphics for Data Analysis [Internet]. Springer-Verlag New York; 2016. Available from: https://ggplot2.tidyverse.org
